# Down‐regulation of Suv39h1 attenuates neointima formation after carotid artery injury in diabetic rats

**DOI:** 10.1111/jcmm.14809

**Published:** 2019-11-17

**Authors:** Jing Zhang, Jian Yang, Changwu Xu, Qi Hu, Jun Hu, Jing Chen, Hong Jiang

**Affiliations:** ^1^ Department of Cardiology Renmin Hospital of Wuhan University Wuhan China; ^2^ Department of Cardiology The First College of Clinical Medical Science China Three Gorges University & Yichang Central People's Hospital Yichang China; ^3^ Central Laboratory The First College of Clinical Medical Science China Three Gorges University & Yichang Central People's Hospital Yichang China

**Keywords:** diabetes mellitus, migration, neointima formation, proliferation, Suv39h1, vascular injury

## Abstract

Patients with diabetes have an increased risk of vascular complications. Suv39h1, a histone methyltransferase, plays a protective role against myocardial injury in diabetes. Herein, we intend to explore whether Suv39h1 could affect neointimal formation after vascular injury in diabetic rats and reveal the underlying mechanism. In this study, we generated adenovirus expressing Suv39h1 as well as lentivirus expressing Suv39h1‐targeting shRNA and evaluated the significance of Suv39h1 in vascular smooth muscle cells (VSMCs) under diabetic conditions*. *In vitro*,* we examined proliferative and migratory behaviours as well as the underlying signalling mechanisms in VSMCs in response to high glucose treatment. In vivo, we induced diabetes in SD rats with streptozocin and established the common carotid artery balloon injury model. Suv39h1 was found to be both necessary and sufficient to promote VSMC proliferation and migration under high glucose conditions. We observed corresponding changes in intracellular signalling molecules including complement C3 and phosphor‐ERK1/2. However, either up‐regulating or down‐regulating Suv39h1, phosphor‐p38 level was not significantly affected. Consistently, Suv39h1 overexpression led to accelerated neointima formation, while knocking down Suv39h1 reduced it following carotid artery injury in diabetic rats. Using microarray analyses, we showed that altering the Suv39h1 level in vivo dramatically altered the expression of myriad genes mediating different biological processes and molecular function. This study reveals the novel role of Suv39h1 in VSMCs of diabetes and suggests its potential role as a therapeutic target in diabetic vascular injury.

## 1 INTRODUCTION

According to the latest data from the International Diabetes Federation (http://www.idf.org), more than 387 million people worldwide are living with diabetes mellitus (DM), and this number is expected to increase to almost 600 million by 2035. Among all chronic complications of type 2 DM, coronary artery disease accounts for the majority of morbidity and mortality.^1^ Atherosclerosis in DM is characterized by diffuse injuries that affect multiple distal coronary branches and accelerated progression, imposing a great challenge for revascularization.^2^, ^3^, ^4^

At the cellular and molecular level, multiple mechanisms contribute to the pathophysiology of DM atherosclerosis. In the first of these mechanisms, hyperglycaemia, an increase in free fatty acids and insulin resistance associated with DM all act in concert on vascular endothelial cells. This leads to endothelial dysfunction and disruption of the intact endothelial barrier and is a key precursor step in the pathogenesis of atherosclerosis.^5^ Upon exposure of the subendothelial matrix to blood circulation, numerous platelet‐activating factors, including thrombin, thromboxane, platelet‐activating factor and collagen contained within the matrix, activate platelets and induce immediate thrombus formation.^6^ This, in turn, generates chemotactic and mitogenic factors to stimulate the migration and proliferation of vascular smooth muscle cells (VSMCs) leading to neointima formation and subsequent vascular restenosis.^7^, ^8^ Currently, the therapy for DM atherosclerosis includes percutaneous coronary intervention and coronary artery bypass graft. However, revascularization efforts are always accompanied by a higher rate of restenosis, delayed side effects and poor long‐term outcomes.^9^ Therefore, it is highly desirable to identify new targets that potentially inhibit neointima formation and simultaneously protect the endothelial barrier from risk factors associated with DM.

Intimal hyperplasia is mainly because of the proliferation and synthesis activation of VSMCs, which is mediated by cell cycle progression.^10^ Cell cycle involves a family of cyclins and their associated kinases, and specific signal transduction mechanism. Cyclin‐dependent kinase inhibitor (CKI) family, including p15 and p16, can bind to almost all cyclin‐dependent kinases (CDKs) and subsequently initiates cell cycle arrest.^11^ Several cellular pathways could regulate the cell cycle. The phosphoinositide‐3 kinase (PI3K)/Akt, Wnt and Nrf2 are well‐known signalling pathways for regulating cell proliferation, especially under diabetic condition.^12^, ^13^, ^14^ Another important signal pathway in response to vascular injury and pertinent to diabetes is mitogen‐activated protein kinases (MAPKs), which contains extracellular signal‐regulated kinase 1/2 (ERK1/2) and p‐38 MAPK.^15^ Activation of MAPK signalling induces cell cycle entry and promotes restenosis progression in diabetic animals.^16^ Moreover, abnormal increase in complement C3 induces the synthetic phenotype in VSMCs,^17^ which is associated with activation of ERK1/2.^18^ However, its role in diabetic vascular remoulding has not been fully investigated.

Histone acetylation and methylation play important roles in cellular pathophysiology and are currently under intense investigations.^19^, ^20^ Suv39h1 is a histone methyltransferase that specifically trimethylates lysine 9 of histone H3 and functions as a transcriptional repressor.^21^ Recent studies revealed that Suv39h1 is an important regulator of gene expression in cycling and non‐cycling cells, and is associated with the development of multiple cancers.^22^, ^23^ In DM, Suv39h1 has been shown to protect VSMCs from metabolic memory and proinflammatory phenotypes.^24^ However, the direct effects of Suv39h1 on DM‐induced VSMC migration and proliferation in vitro or neointima formation in vivo remain largely elusive. Therefore, we intend to explore whether Suv39h1 plays a regulatory role in neointimal hyperplasia after vascular injury in diabetic rat via modulation on CKI or MAPK activation. In this study, we applied both gain‐of‐function and loss‐of‐function approaches by generating an adenovirus with Suv39h1 overexpression and a lentivirus with Suv39h1 silence, respectively. We investigated the functional significance and underlying mechanisms of Suv39h1 in VSMC migration and proliferation in vitro in response to high glucose treatment as well as neointima formation in vivo following arterial injury in diabetic rats.

## 2 MATERIALS AND METHODS

### 2.1 Experimental animals

All animal experimental protocols were approved by the Institutional Animal Care and Use Committee of Wuhan University and conformed to the Guide for the Care and Use of Laboratory Animals by the National Institutes of Health. Male, specific pathogen‐free Sprague Dawley (SD) rats (140‐150 g) were provided by the Experimental Animal Center of Wuhan University (Wuhan, China). Animals were housed individually in standard polypropylene cages at controlled temperature (22 ± 2°C) and humidity (55 ± 5%) with a 12:12‐hour light‐dark cycle and with free access to water and a normal chow diet.

The rat model of diabetes was established as previously described.^25^, ^26^ To induce diabetes mellitus, all rats were fed with high‐fat diet (HFD, 60% fat) for 4 weeks. Subsequently, the rats received a single intraperitoneal injection of streptozotocin (STZ, Sigma) at 40 mg/kg followed by resumption of an HFD for a further 2 weeks. Three days after the STZ injection, rats with non‐fasting blood glucose levels more than 15 mmol/L were considered diabetic and used for later experiments. Control rats (Nor rats) were fed a normal chow diet.

To induce vascular injury, the rat carotid artery balloon injury model was established at 2 weeks after STZ injection as reported previously.^27^ Immediately after injury, normal saline (vehicle control), adenovirus (5 × 10^8^ PFU/mL) or lentivirus (10^8^ TU/mL) was inoculated into the damaged vessels. After local incubation of the virus for 20 minutes, the clamps on the common and internal carotid arteries were released to restore blood flow. The lesion was cleaned and sutured by layers.

Seven days (for RT‐PCR and microarray analyses) or 28 days (for histological analysis) after the balloon injury and viral infection, the rats were killed by an overdose of isoflurane. The injured common carotid artery was isolated, excised and cut into segments.

### 2.2 Production of Suv39h1‐overexpressing adenovirus and Suv39h1‐knocking down lentivirus

An adenoviral vector carrying the rat Suv39h1 gene was constructed by Vector Gene Technology Company Limited. To down‐regulate Suv39h1 expression, four shRNA oligonucleotides targeting different regions of the Suv39h1 gene were designed, synthesized and cloned into the lentiviral plasmid GV118 from GeneChem as described previously.^28^

### 2.3 Isolation of VSMCs and treatments

Primary VSMCs were isolated from rat aorta by a modification of the explant technique and cultured in vitro as previously described.^29^, ^30^ VSMCs growing in log phase between passages three and five were used in this study.

For adenoviral infection, isolated VSMCs in log‐phase growth were infected with either Suv39h1 adenoviruses (Ad‐Suv39h1) or control adenoviruses (Ad‐Null) (MOI = 15) for 4 hours and then cultured in Dulbecco's modified Eagle's medium containing 10% foetal bovine serum (FBS), 100 U/mL penicillin and 100 μg/mL streptomycin (HyClone) for 3 days before any further treatments. For lentiviral infection, isolated VSMCs in log‐phase growth were infected with lentivirus vectors containing either Suv39h1 shRNA sequence (LV‐Suv39h1) or control shRNA sequence (LV‐NC) (MOI = 40) together with polybrene (5 μg/mL) for 12 hours and then cultured in regular growth medium described above for 5 days before any further treatments. Cells were incubated at 37°C in a humidified atmosphere of 95% air and 5% CO_2._ VSMCs were stimulated with 5 mmol/L glucose (normal glucose, NG), 5 mmol/L glucose + 25 mmol/L mannitol (osmotic control, MN) and 30 mmol/L glucose (high glucose, HG) as previously described.^31^

### 2.4 Western blot analysis

Total protein was extracted from cells using RIPA buffer (Beyotime), with an equal amount loaded onto 12% SDS‐PAGE gel. The following primary antibodies were used for Western blot analysis: Suv39h1 (48 kD, 1:800; Abcam, ab12405), proliferating cell nuclear antigen (PCNA, 29 kD, 1:300; Bioss, bs‐0754R), complement C3 (180 kD, 1:200; Santa Cruz, sc20137, CA), phosphor‐p38 (Tyr323) (41 kD, 1:300; Bioss, bs‐5477R), phosphor‐ERK1/2 (Thr202 + Tyr204) (41 kD, 1:300; Bioss; bs‐3016R), p38 (41 kD, 1:300; Bioss, bs‐0637R), ERK1/2 (42/44 kD, 1:300; Bioss, bs‐2637R), histone H3K9 trimethylation (H3K9me3, 15.4 kD, 1:800; Abcam, ab8898) and GAPDH (37 kD, 1:1000; Cell Signaling, #5174). Following the incubation with primary antibodies, HRP‐conjugated secondary antibodies (1:1500‐1:2000; Boster) were used. The primary antibody was diluted with Tris‐buffered saline Tween‐20 (TBST) containing 5% skimmed milk powder.

### 2.5 Quantitative RT‐PCR analysis

Total RNA was extracted using TRIzol (Invitrogen) and was reverse‐transcribed into cDNA using SuperScript™ III Reverse Transcriptase (Invitrogen) following the manufacturer's instructions. The cDNA was then quantified using real‐time PCR (RT‐PCR, Applied Biosystems) under the following conditions: denaturation at 95°C for 10 minutes, followed by 40 cycles of 95°C for 10 seconds and 60°C for 60 seconds. The primer sequence for RT‐PCR is summarized in Table S1. The data analysis for RT‐PCR was performed with the 2^−ΔΔCt^ method.

### 2.6 Cell proliferation assay

Cell proliferation was assessed using the CCK‐8 kit (Dojindo Laboratories). First, 8 × 10^3^ VSMCs were seeded in each well of a 96‐well plate and cultured for 24 hours in medium. After synchronization with Dulbecco's modified Eagle's medium containing 0.5% foetal bovine serum for 24 hours, VSMCs were stimulated with 30 mmol/L glucose for an additional 24 hours. Following CCK‐8 treatment, OD values were determined at 450 nm using a microplate spectrophotometer (Bio‐Rad).

### 2.7 Cell migration assays

The transwell migration assay was performed as previously described.^19^ Briefly, 1 × 10^5^ VSMCs were loaded on the upper chamber of a cell culture insert. Glucose was added to the lower well. After 16 hours, the transwell insert was removed from the plate. A cotton‐tipped applicator was used to carefully remove the non‐migrating cells from the top of the membrane without damaging it. After methanol fixation, the transwell insert was stained with 0.1% crystal violet. The migrated cells were imaged under an inverted microscope (100×), and the number of cells counted from five different fields of view (upper, lower, right, left and centre) was used to obtain an average sum of cells that migrated through the membrane towards glucose.

### 2.8 ChIP assay

ChIP assays were performed with the ChIP kit (Millipore) as previously described.^31^ Briefly, VSMCs were grown to 60% confluence and then infected with LV‐NC and LV‐Suv39h1 for 5 days. After stimulation with HG for 16 hours, the cells were washed and cross‐linked with 1% formaldehyde. Subsequently, the cross‐linking was stopped using 1.25 mol/L glycine. The cell lysates were then collected and subjected to immunoprecipitation with H3K9me3 antibody or IgG (Santa Cruz). Protein‐DNA cross‐links were reversed, and the DNA was extracted. ChIP‐enriched DNA samples were analysed by real‐time qPCR using primer sequences near the promoter sites on C3, p15 and p16. The data were analysed using the 2^‐∆∆Ct^ method and normalized with input samples. The results were expressed as fold changes relative to the LV‐NC group. The primer sequences used were as follows:

C3: forward 5′‐AAAGTCTGCCGTGGTCATTC‐3′;

reverse 5′‐GGGAGTGGGGTGGTTAAGC‐3′.

p15: forward 5′‐GCCTTGAGATGAACGAGCCT‐3′;

reverse 5′‐CTGAAAGAAAGCCTTTAGGGTG‐3′.

p16: forward 5′‐GTTTGTGAGAGCAGGGATTTC‐3′;

reverse 5′‐CTTGGTTGTTTGTAGGGAGATT‐3′.

### 2.9 Histological analysis

The segments of injured common carotid artery at 28 days after the balloon injury and viral infection were fixed in 4% paraformaldehyde and embedded in paraffin to generate 5‐µm sections. The sections were then stained with haematoxylin and eosin (H&E) for imaging under microscope, and the medial area (MA) and intimal area (IA) were measured using the Image‐Pro Plus 6.0 image analysis system (Fryer Co).

To examine the expression and distribution of PCNA and CD31 in injured vessels, the sections were de‐paraffinized, followed by antigen retrieval using a microwave. After staining with anti‐PCNA or anti‐CD31 primary antibody followed by HRP‐conjugated secondary antibody, the sections were developed using DAB substrate. The stained sections were imaged under a microscope (200×) with the number of positively stained cells quantified from five random fields.

To evaluate collagen generation after balloon injury, Masson's trichrome staining was also performed, and the ratio of collagen area was calculated (the ratio of collagen area = collagen area/(collagen area + muscle area) × 100%).

### 2.10 Microarray analysis

On day 7 after the balloon injury and viral infection, segments of injured common carotid arteries were collected, stored in liquid nitrogen and sent to KangChen Biotech (Shanghai, China) for microarray analysis. Microarray analyses were performed with the Affymetrix system as previously described.^32^ Briefly, the data were first analysed using the MAS 5.0 normalization method of the Affymetrix Software. The log_2_‐transformed expression values were then normalized across samples by *Z*‐score calculations using Spotfire DecisionSite for Microarray Analysis. Analysis of variance (ANOVA) was used to identify genes whose expression was significantly different (*P* < .05). Gene enrichment analyses were carried out using GeneGO (http://www.genego.com/) gene ontology software.

### 2.11 Statistical analysis

All statistical analysis was conducted using SPSS 17.0 software. Quantitative data were presented as means ± SD. Student's *t* test was used for comparisons between two groups, and one‐way ANOVA was used for multiple comparisons. A *P* < .05 was considered statistically significant.

## 3 RESULTS

### 3.1 Suv39h1 is necessary and sufficient for VSMC migration and proliferation in response to high glucose treatment

**Figure 1:**
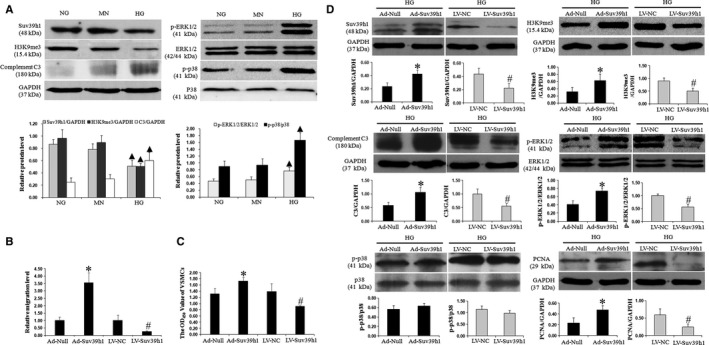
Suv39h1 is an essential regulator of the migratory and proliferative behaviours of isolated VSMCs. Isolated VSMCs were treated with NG, MN or HG. The endogenous expression of Suv39h1, H3K9me3, complement C3, p‐ERK1/2, ERK1/2, p‐p38 and p38 were detected by Western blot and quantified from four independent experiments (A, n = 4). VSMCs were infected with either Ad‐Suv39h1 vs Ad‐Null or LV‐Suv39h1 vs LV‐NC under HG stimulation. Migration and proliferation were detected by transwell migration assay (B, n = 5) and CCK‐8 kit (C, n = 6). The expressions of molecules, including H3K9me3, complement C3, p‐ERK1/2, ERKE1/2, p‐p38, p38 and PCNA (D, n = 4), were detected by Western blot and quantified as the ratio to internal control protein GAPDH (relative protein level). ^▲^ *P* < .05, as compared to the NG group. *P < .05, as compared to the Ad‐Null group; ^#^ *P* < .05, as compared to the LV‐NC group

We first examined the response of endogenous Suv39h1 in response to high glucose treatment (Figure 1A). We found the endogenous Suv39h1 and H3K9me3 were both significantly reduced by almost 50%, respectively (*P* < .05, as compared to normal glucose treatment). At the endogenous level, in contrast to the down‐regulation of Suv39h1 in response to HG, complement C3 level increased by approximately onefold, which was followed by activation of p38 and ERK1/2 phosphorylation with significant differences, respectively. However, the additional concentration of mannitol did not affect protein expression of Suv39h1, H3K9me3, complement C3 and phosphorylated protein level of ERK1/2 and p38. Next, to understand the biological significance of Suv39h1 in diabetic vascular injury, we examined its role in the migration and proliferation of VSMCs cultured in vitro, two critical behaviours in response to diabetic vascular injury*.* To this end, we used both gain‐of‐function and loss‐of‐function approaches, and either overexpressed Suv39h1 by Ad‐Suv39h1 infection or knocked down the endogenous Suv39h1 by LV‐Suv39h1. Following high glucose treatment, Ad‐Suv39h1‐infected VSMCs exhibited a significantly higher migratory capability than Ad‐Null‐infected cells (*P* < .05). In contrast, knocking down Suv39h1 by LV‐Suv39h1 led to dramatically reduced migration compared with cells infected with the control lentivirus (LV‐NC, *P* < .05; Figure 1B). When examining the effects on cell proliferation using CCK‐8 kit, we found that Suv39h1 overexpression increased the value of OD_450_ more than 30% in response to HG for 24 hours, while knocking down Suv39h1 showed the opposite effect (both *P* < .05; Figure 1C). Moreover, we found that Ad‐Suv39h1 infection increased the PCNA level by more than onefold, while LV‐Suv39h1 shRNA reduced the PCNA level by approximately 50%, as compared with control virus infection (*P < *.05, Figure 1D).

### 3.2 Suv39h1 modulates the expressions of multiple downstream signalling molecules in VSMCs in response to high glucose

As shown in Figure 1D, Suv39h1 expression level was markedly elevated in VSMCs after Ad‐Suv39h1 infection for 3 days, but not in VSMCs infected with Ad‐Null. Compared with the LV‐NC group, a significant reduction in Suv39h1 was observed in VSMCs infected with LV‐Suv39h1. To understand the molecular mechanism by which Suv39h1 modulates high glucose‐induced cell migration and proliferation, we focused on the downstream signalling molecules for these stresses including complement C3, phosphor‐ERK1/2 and phosphor‐p38.

**Figure 2:**
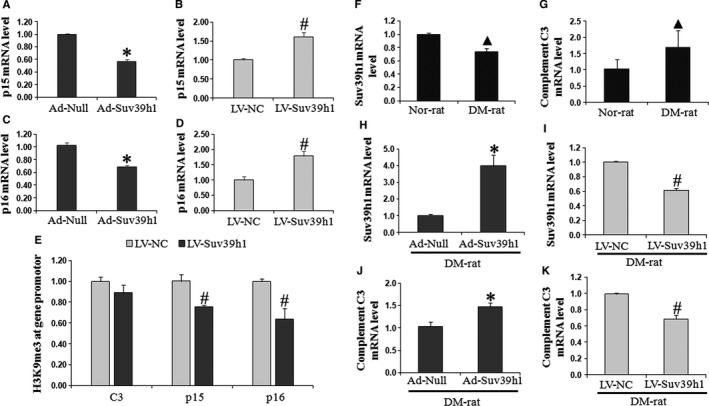
Suv39h1 is the key regulator of complement C3, p15 and p16 induced by HG. The mRNA expressions of p15 and p16 were tested by RT‐PCR after transfection of Ad‐Suv39h1, Ad‐Null, LV‐Suv39h1 or LV‐NC (A‐D, n = 3). H3K9me3 levels at the promoters of complement C3, p15 and p16 in VSMCs were quantified by Chip‐PCR (E, n = 3). Moreover, Suv39h1 critically regulates the expression of complement C3 in diabetic vascular injury in vivo*.* Rats were fed with HFD for 4 weeks and received a single intraperitoneal injection of STZ at 40 mg/kg followed by resumption of HFD for a further 2 weeks. Carotid artery balloon injury model was established at 2 weeks after STZ injection. On day 7 after establishing the carotid artery balloon injury model and infecting the injured vessels locally with normal saline (NS; F, G), Ad‐Suv39h1 vs Ad‐Null or LV‐Suv39h1 vs LV‐NC, the injured vessels were excised, and the steady‐state mRNA levels of Suv39h1 and complement C3 were detected by RT‐PCR (from H‐K, n = 3). Three carotid arteries were pooled in each separate experiment. Nor‐rat: non‐diabetic rats undergoing carotid artery balloon injury and local inoculation of NS; DM‐rat: diabetic rats undergoing carotid artery balloon injury and local inoculation of NS. ^▲^ *P* < .05, as compared to the Nor‐rat group; *P < .05, as compared to the Ad‐Null group; ^#^ *P* < .05, as compared to the LV‐NC group

Overexpressing Suv39h1 significantly increased the level of complement C3 and phosphor‐ERK1/2 in response to high glucose treatment (*P* < .05, as compared to Ad‐Null). Moreover, 20 μmol/L PD98059 (an inhibitor of ERK1/2, Sigma, MO) pre‐treatment partially neutralized VSMC proliferation and migration induced by Suv39h1 overexpression (Figure S1), which suggested ERK1/2 activation plays a critical role in Suv39h1 action on HG‐induced VSMCs. On the other hand, knocking down Suv39h1 reduced the level of complement C3 and phosphor‐ERK1/2 in response to high glucose treatment (*P* < .05, as compared to LV‐NC). By either up‐regulating or down‐regulating Suv39h1, phosphor‐p38 level was not significantly affected (Figure 1D). Meanwhile, the cyclin‐dependent kinase inhibitor p15 and p16 mRNA levels were increased by 60% and 70% in the LV‐Suv39h1 group when compared with the LV‐NC group, respectively. However, Suv39h1 overexpression displayed the opposite effect (Figure 2A‐D). Furthermore, in order to explore the potential mechanism of Suv39h1 on VSMCs’ pathological activation, we found that the H3K9me3 levels on the p15 and p16 promoters were decreased by Suv39h1 silencing, which would subsequently promote transcription of the latter two (Figure 2E). Nevertheless, there was no significant difference in H3K9me3 level on the C3 promotor (*P* > .05, as compared to LV‐NC).

### 3.3 Down‐regulation of Suv39h1 attenuates neointima formation and promotes re‐endothelialization after carotid artery injury in diabetic rats

Following the in vitro characterization of Suv39h1 in isolated VSMCs, we moved to the well‐established carotid artery balloon injury model. Immediately following injury, we inoculated Ad‐Suv39h1 vs Ad‐Null or LV‐Suv39h1 vs. LV‐NC locally into the injured vessels. The efficiency of local virus infection was optimal 7 days after injury, which met the needs of subsequent experiments (Figure S2). Moreover, random blood glucose was significantly enhanced after HFD combined with STZ induction, when compared with the normal control rats (Table S2).

To examine the endogenous alterations of Suv39h1 and its targets in response to vascular injury in diabetic rats, we first quantified the steady‐state mRNA level of Suv39h1 and complement C3 in injured vessels. As shown in Figure 2F, vascular injury decreased the expression of endogenous Suv39h1, but increased that of complement C3 from in vivo injured vessels, consistent with the in vitro observation in isolated VSMCs in response to HG (Figure 2G). Ad‐Suv39h1 local infection increased the expression of Suv39h1 by approximately 3 times (*P* < .05, as compared to Ad‐Null; Figure 2H), while LV‐Suv39h1 reduced it by almost 40% in the injured vessels (*P* < .05, as compared to LV‐NC; Figure 2I). By quantifying the expression of complement C3 in injured vessels following viral inoculation, we found that ectopic expression of Suv39h1 was sufficient to up‐regulate complement C3 (*P* < .05, as compared to Ad‐Null), while knocking down Suv39h1 reduced it in injured vessels (*P* < .05, as compared to LV‐NC; Figure 2J, K). For the arteries without balloon injury in diabetic mice, the expression of complement C3 has not significantly changed in Ad‐Suv39h1 or in LV‐Suv39h1 group when compared with their corresponding control group (Figure S3).

**Figure 3:**
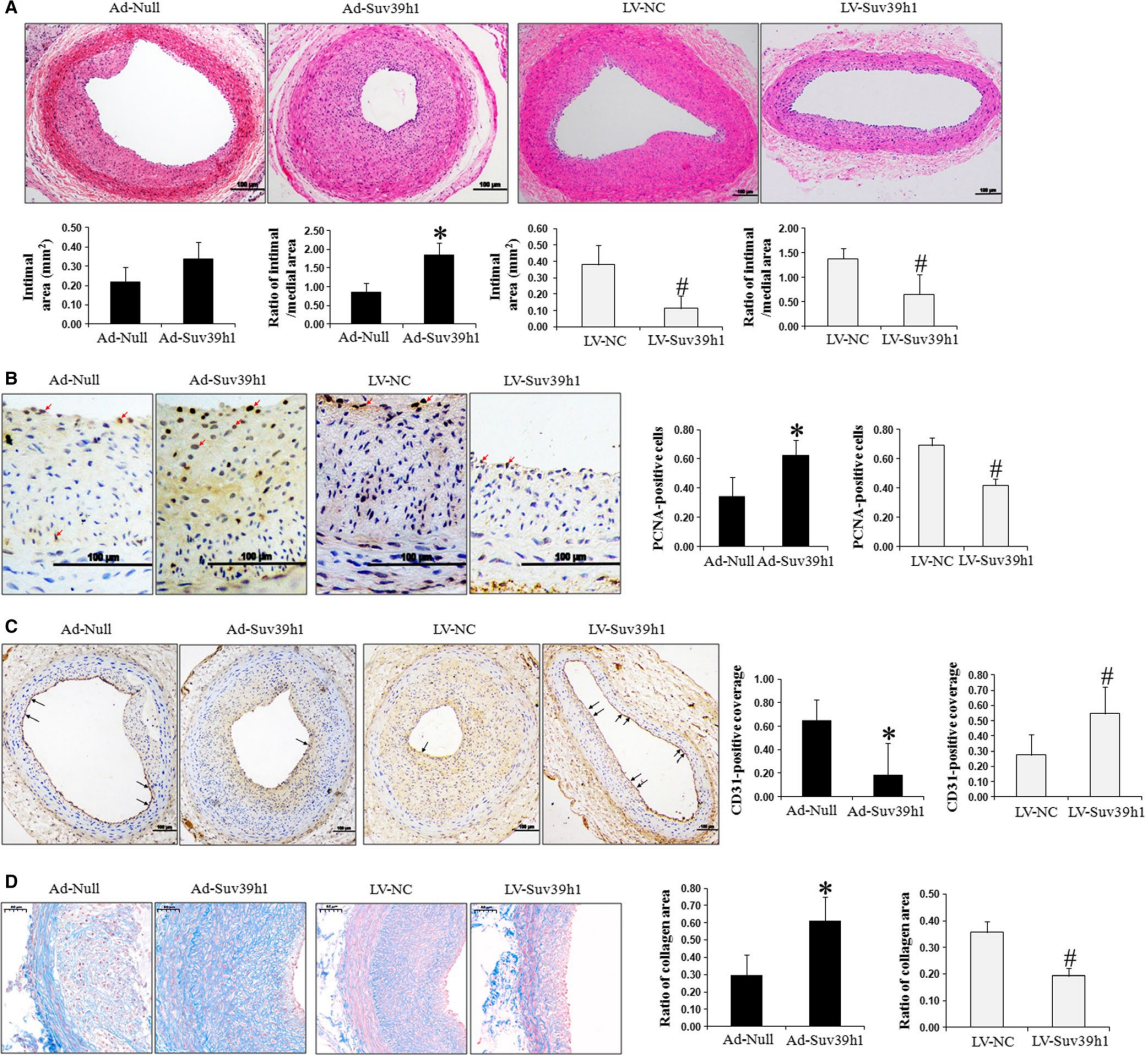
Suv39h1 promotes neointima formation, but inhibits the re‐endothelialization of injured vessels in diabetic rats. A, On day 28 after establishing the carotid artery balloon injury model and infecting the injured vessels locally with Ad‐Suv39h1 vs Ad‐Null or with LV‐Suv39h1 vs LV‐NC, the injured vessels were isolated and stained with HE to measure medial area (MA) and intimal area (IA), as well as to calculate the I/M ratio. Representative images from each group are shown at the top and the quantification of IA and I/M ratio at the bottom. The proliferative activity (B) and re‐endothelialization (C) within the neointima were detected by immunohistochemical staining of PCNA and CD31, respectively. The representative image from each group is shown on the left (scale bar: 100 µm), and quantification of the average percentage of PCNA^+^ cells from five random fields or the rate of CD31^+^ coverage cells is displayed on the right. The red arrows indicate PCNA^+^ cells. The black arrows indicate CD31^+^ endothelium. Representative Masson's trichrome‐stained photomicrographs of carotid arteries from different groups were also performed (scale bar: 50 µm, D). Collagen was stained blue. *P < .05, as compared to the Ad‐Null group; ^#^ *P* < .05, as compared to the LV‐NC group (n = 6)

Functionally, we observed enhanced neointima formation following local infection with Ad‐Suv39h1. Figure 3A showed an increasing trend in IA (*P > *.05) and a significant increase in the Ad‐Suv39h1 group's IA/MA (I/M) ratio (*P* < .05) as compared to the Ad‐Null group. In addition, knocking down Suv39h1 by LV‐Suv39h1 infection led to a significant reduction of neointima formation, as measured by IA and the I/M ratio (both *P* < .05, as compared to LV‐NC).

To examine underlying changes in vascular proliferation and re‐endothelialization in response to diabetic vascular injury, we quantified the percentage of PCNA^+^ cells (Figure 3B) and that of CD31^+^ cells within the neointima on day 28 after the procedure (Figure 3C). We found that Suv39h1 overexpression increased the percentage of PCNA^+^ cells by approximately onefold (*P* < .05, as compared to Ad‐Null), while inhibiting Suv39h1 significantly reduced the percentage of PCNA^+^ cells (*P* < .05, as compared to LV‐NC). In contrast, Suv39h1 overexpression significantly reduced re‐endothelialization as measured by the percentage of CD31^+^ cells within the neointima, while Suv39h1 reduction enhanced it (*P < *.05, as compared to the corresponding control group).

In addition, Masson's staining was used to detect collagen formation after diabetic vascular injury. As the results shown in Figure 3D, the collagen area was nearly 2.1‐fold larger compared with Ad‐Null when Suv39h1 was overexpressed, while knocking down Suv39h1 had the opposite effect.

### 3.4 Suv39h1 controls the genes underlying multiple biological processes

**Figure 4:**
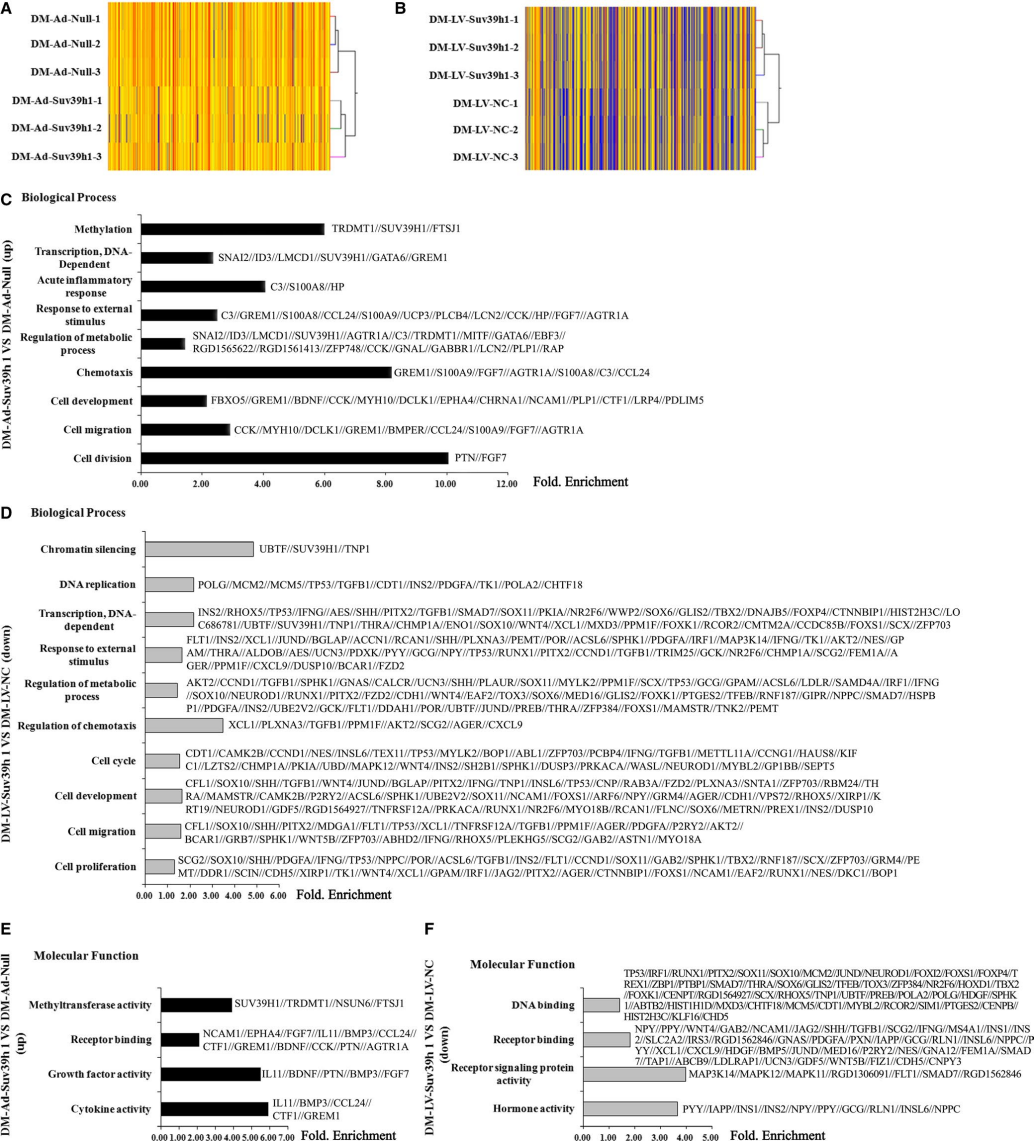
Altering Suv39h1 expression leads to altered genes controlling different biological processes in diabetic injured vessels. A, B, Heat map depicting the gene expression profiles in injured carotid artery of diabetic rats following local inoculation of either Ad‐Suv39h1 (as compared to Ad‐Null, left panel) or LV‐Suv39h1 (as compared to LV‐NC, right panel). Owing to the limited volume, the injured artery segments pooled from three individual mice were integrated into one sample. Three samples from each group were analysed by microarray analysis. GO analysis revealed the distribution of differentially expressed genes by altering vascular Suv39h1 among different biological processes (C, D) and different molecular functions (E, F)

Given that Suv39h1 is a histone methyltransferase that potentially regulates myriad genes simultaneously, we characterized the overall gene expression profile following either Suv39h1 overexpression or knockdown by microarray analysis (Figure 4A, B; Table S3 and Table S4). Owing to the limited volume, three carotid arteries were integrated into one sample. By comparing three samples from each group and including only those genes showing more than 1.5‐fold difference in mRNA levels between two groups as well as *P* < .05, we identified 233 up‐regulated and 221 down‐regulated genes between the Ad‐Suv39h1 and Ad‐Null groups, as well as 849 up‐regulated and 658 down‐regulated genes between the LV‐Suv39h1 and LV‐NC groups. Further Gene Ontology (GO) analysis of these differentially expressed genes showed that they had roles not only in diverse biological processes such as metabolism, cell differentiation, cell development and response to external stimulus (Figure 4C, D), but also in various molecular functions including receptor binding, methyltransferase activity, DNA binding and hormone activity, among others (Figure 4E, F).

## 4 DISCUSSION

In this study, we identified Suv39h1 as a critical regulator of VSMC migration and proliferation in vitro in response to diabetic stimuli and also as a regulator of neointima formation in vivo following vascular injury in diabetic rats. Suv39h1 up‐regulation is sufficient to promote these processes, while Suv39h1 silencing inhibits them. Mechanistically, we showed that the actions of Suv39h1 are mediated via multiple downstream signalling molecules. In response to high glucose levels, Suv39h1 may act on complement C3 to regulate the phosphorylation/activation of ERK1/2. The potential epigenetic mechanism of Suv39h1 action occurs by affecting downstream p15 and p16 expression via H3K9me3 modulation.

The complement system is activated by three pathways: the classical pathway, the alternative pathway and the lectin pathway. The three pathways converge at the point of C3 convertase formation. Complement C3 plays an essential role in normal physiology. Abnormal complement C3 expression or activity leads to multiple diseases such as glomerulonephritis,^33^ acute renal transplant rejection^34^ and atherosclerosis.^35^ In spontaneously hypertensive rats, gene expression analysis and further functional studies revealed complement C3 as stimulating the synthetic phenotype and excessive growth of VSMCs.^36^ Further studies in apoE‐null mice and hypercholesterolaemic patients also have suggested a positive correlation between complement C3 and VSMC proliferation, as well as the role of ERK1/2 activation in this process.^18^ These studies prompted us to examine the role of complement C3 in Suv39h1‐induced VSMC proliferation. We revealed a consistent relationship between altered Suv39h1 expression and complement C3 levels in VSMCs in response to high glucose treatment. Similar changes were also observed for activated ERK1/2 (p‐ERK1/2), but not with activated p38, suggesting that Suv39h1 stimulates VSMC proliferation via control of complement C3 and subsequent ERK1/2, but not by p38 activation. In addition, previous study has proved Suv39h1 could enhance peptidyl‐prolyl cis‐trans isomerase never‐in‐mitosis A (NIMA)‐interacting 1 (PIN1) promoter activity to up‐regulate PIN1 protein level, which induces an increase in direct interaction between PIN1 and MEK1, and results in ERK phosphorylation.^37^, ^38^ Therefore, Suv39h1 could regulate ERK1/2 phosphorylation possibly via complement C3 and PIN1.

Previous studies have confirmed that p15 and p16 are important factors regulating VSMC proliferation.^39^, ^40^ Suv39h1 down‐regulation would promote p15 and p16 expression by inhibiting H3K9me3 in the promoter region, leading to inhibition of VSMC proliferation under pathological stimulation. Unfortunately, there was no significant difference in H3K9me3 levels in the promoter of complement C3, which indicated that direct histone methylation was not the main mechanism of Suv39h1’s effect on complement C3. Instead, one possible mechanism is that Suv39h1 depletion suppresses H3K9me3 in the promoter of peroxisome proliferator–activated receptor γ (PPARγ),^41^, ^42^ leading to an increase in PPARγ expression, which then down‐regulates complement C3 levels indirectly.^43^

Therefore, we hypothesize that Suv39h1 regulates VSMC proliferation in two ways. Firstly, Suv39h1 knockdown attenuates complement C3/ERK1/2 activation, which leads to a change of downstream molecules relative to cell proliferation, including p15^44^ and p16.^45^ Secondly, the down‐regulation of Suv39h1 results in the reduction of H3K9me3 in p15 and p16 promoters, which increases expression of the latter two, leading to inactivation of CDKs.

Consistent with the observations in vitro, abnormal blood glucose metabolism was the main manifestation in this animal model (Table S2). Meanwhile, we observed a similar correlation between altered Suv39h1 levels and complement C3 from injured carotid arteries in diabetic rats. In addition to these two molecules, we identified changes in hundreds of genes with various functions involved in distinct biological processes, such as 42 cell proliferation genes (including complement C3), 35 cell cycle genes and 11 DNA replications genes that were all down‐regulated by knocking down Suv39h1. The down‐regulation of these genes together with complement C3/p‐ERK1/2 signalling may contribute to reduced neointimal formation by VSMCs. In contrast, Suv39h1 overexpression enhanced the expression of genes involved in receptor binding, methyltransferase activity, growth factors and cytokine activities, including fibroblast growth factor 7 (FGF‐7) and angiotensin II type 1a receptor (AGTR1A), both of which have been shown to promote the in vitro proliferation of VSMCs.^46^, ^47^ The differential gene expression profiles identified in the injured aorta of Ad‐Suv39h1‐ vs Ad‐Null‐infected or in LV‐Suv39h1‐ vs. LV‐NC‐infected diabetic rats provide novel mechanistic insights into Suv39h1‐mediated regulation of VSMC behaviour in diabetes. Consistent with the role of differential gene regulation, we showed that Suv39h1 overexpression promotes neointima formation as well as its inhibition following the knockdown of Suv39h1.

In previous study, Suv39h1 overexpression plays a negative role in inflammatory genes of *db/db* VSMCs, including interleukin‐6, macrophage colony‐stimulating factor and monocyte chemoattractant protein‐1, via increasing H3K9me3 modification on promoters^24^. These results suggest that Suv39h1 overexpression inhibits the inflammatory response in the diabetic arteries, which would protect VSMCs from metabolic memory and proinflammatory phenotypes. This is in contrast to the findings that Suv39h1 overexpression deteriorates neointimal hyperplasia under diabetic condition. It would suggest that the decreased inflammatory response in Suv39h1 overexpression, by itself, is not sufficient to attenuate neointimal formation after vascular injury. Moreover, the repressive effect of Suv39h1 on cell cycle suppressor, including p15 and p16, plays more important role than proinflammatory phenotypes in VSMC pathological activation, which is consistent with previous studies in cancer.^48^

Interestingly, we found that in response to HG treatment in vitro and artery injury in vivo, the endogenous Suv39h1 level was mildly reduced (though not as much as the reduction achieved by Suv39h1 shRNA knockdown), while complement C3 level increased. The down‐regulation of endogenous Suv39h1 may be a negative feedback loop inhibiting VSMC proliferation, neointima formation and re‐endothelialization in order to repair vascular damage. The opposite regulation between Suv39h1 and complement C3 suggests the role of multiple regulators controlling complement C3 levels in vivo. A minor reduction of Suv39h1 is not sufficient to inhibit these molecules, which may require other positive regulators resulting in their up‐regulation. Functionally, this result is consistent with the observation that the vascular injury in diabetic patients is associated with higher level of neointima formation, as the antagonizing activity of Suv39h1 cannot be accomplished by down‐regulating complement C3. A more dramatic reduction of Suv39h1, such as that achieved by shRNA, however, plays a dominant role in down‐regulating the molecule.

In addition to the action on neointima formation, re‐endothelialization, as measured by percentage of CD31^+^ cells, was negatively regulated by Suv39h1 level. Vascular endothelial cells (ECs) are important components of the vascular structure and have important physiological functions. In addition to functioning as the vascular barrier, these cells also produce a great variety of bioactive molecules, regulating vascular tone, cell adhesion, VSMC proliferation and vessel wall inflammation.^49^ Following vascular injury in diabetes, enhanced apoptosis and reduced regeneration of ECs lead to delayed re‐endothelialization and impaired endothelial functioning.^50^ Current interventional approaches for diabetic vascular injury, such as drug‐eluting stents, effectively inhibit VSMC‐mediated neointima formation as well as the proliferation of ECs, leading to delayed vessel healing and an increased risk of thrombotic complications.^51^ In this study, we showed that knocking down Suv39h1 stimulates re‐endothelialization while inhibits neointima formation. Although the underlying mechanism is still unclear, we propose that the down‐regulation of complement C3 may contribute to this phenotype. A reduced level of complement C3 decreases its deposition on the EC surface, reducing complement‐induced membrane attack on ECs and subsequent EC death.^52^, ^53^

## 5 CONCLUSIONS

Targeting Suv39h1 not only inhibits VSMC migration and proliferation as well as neointima formation, but it also promotes re‐endothelialization after vascular injury in diabetes. Therefore, Suv39h1 may constitute a promising therapeutic target in vascular complications involving diabetes mellitus.

## CONFLICT OF INTEREST

The authors confirm that there are no conflicts of interest.

## AUTHORS’ CONTRIBUTIONS

Jing Zhang conceived of the study, wrote and reviewed manuscript. Jian Yang performed molecular examinations. Changwu Xu performed histological and statistical analysis. Qi Hu and Jun Hu performed animal surgery and collected the data. Jing Chen made data analysis and revised the manuscript. Hong Jiang contributed to the interpretation of the results and critical revision of the manuscript for important intellectual content. All authors read and approved the final manuscript.

## Data Availability

The data that support the findings of this study are available from the corresponding author upon reasonable request.
