# Supplementary figures and images for: Down‐regulation of Suv39h1 attenuates neointima formation after carotid artery injury in diabetic rats
Source: J Cell Mol Med. 2019 Nov 17;24(1):973–83. doi: 10.1111/jcmm.14809 (PMC6933362; doi:10.1111/jcmm.14809)

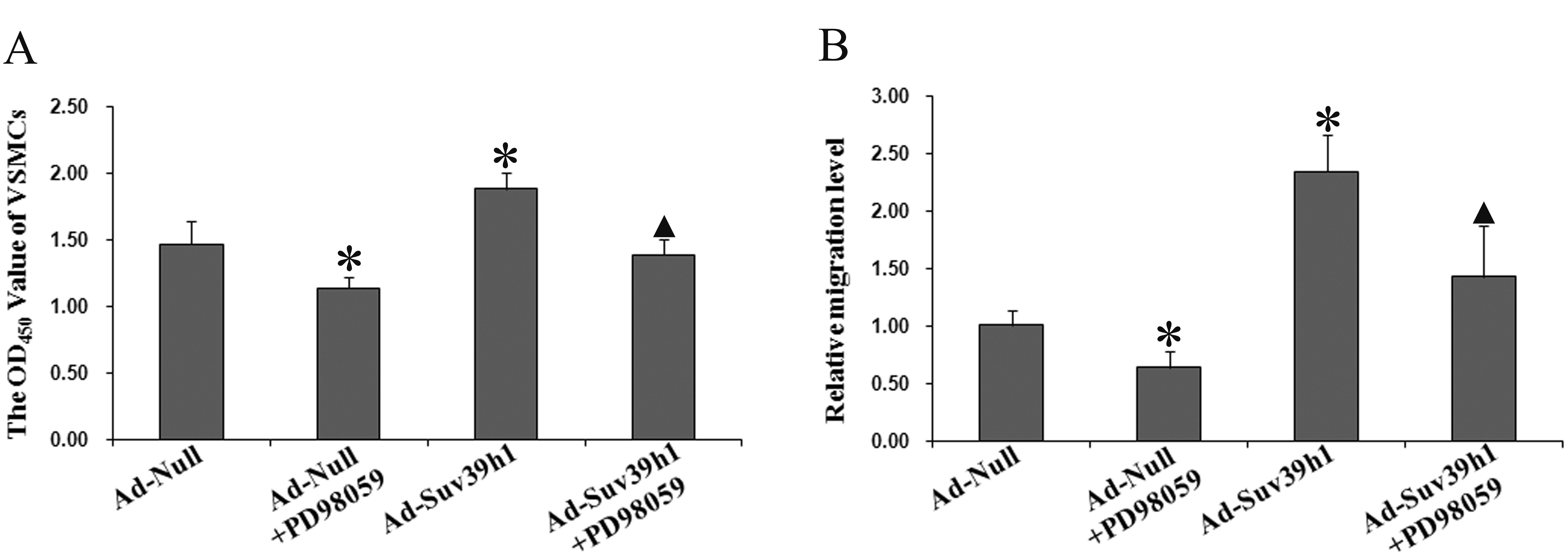

Supplement: Supplementary file 1 [file JCMM-24-973-s001.tif]

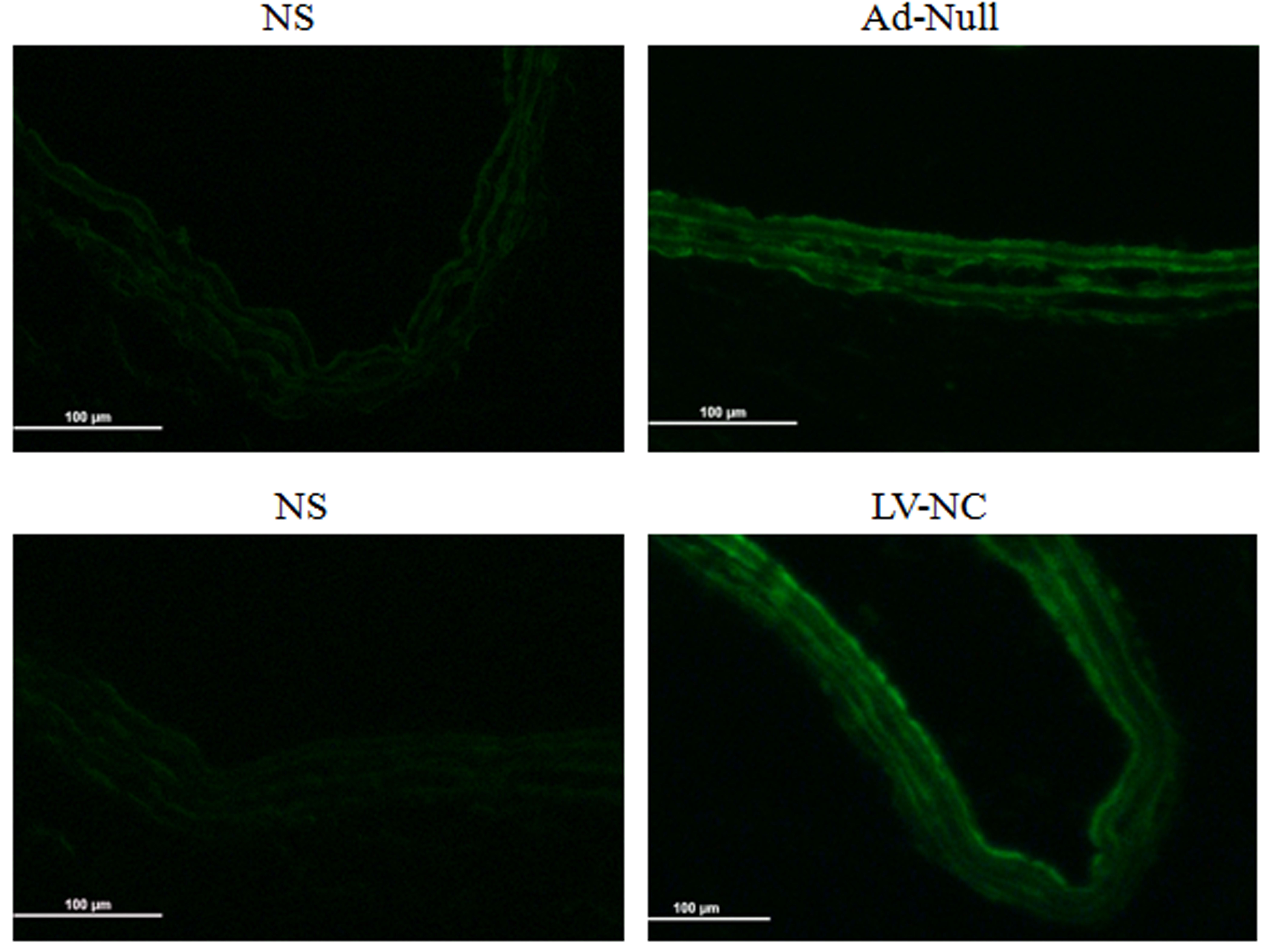

Supplement: Supplementary file 2 [file JCMM-24-973-s002.tif]

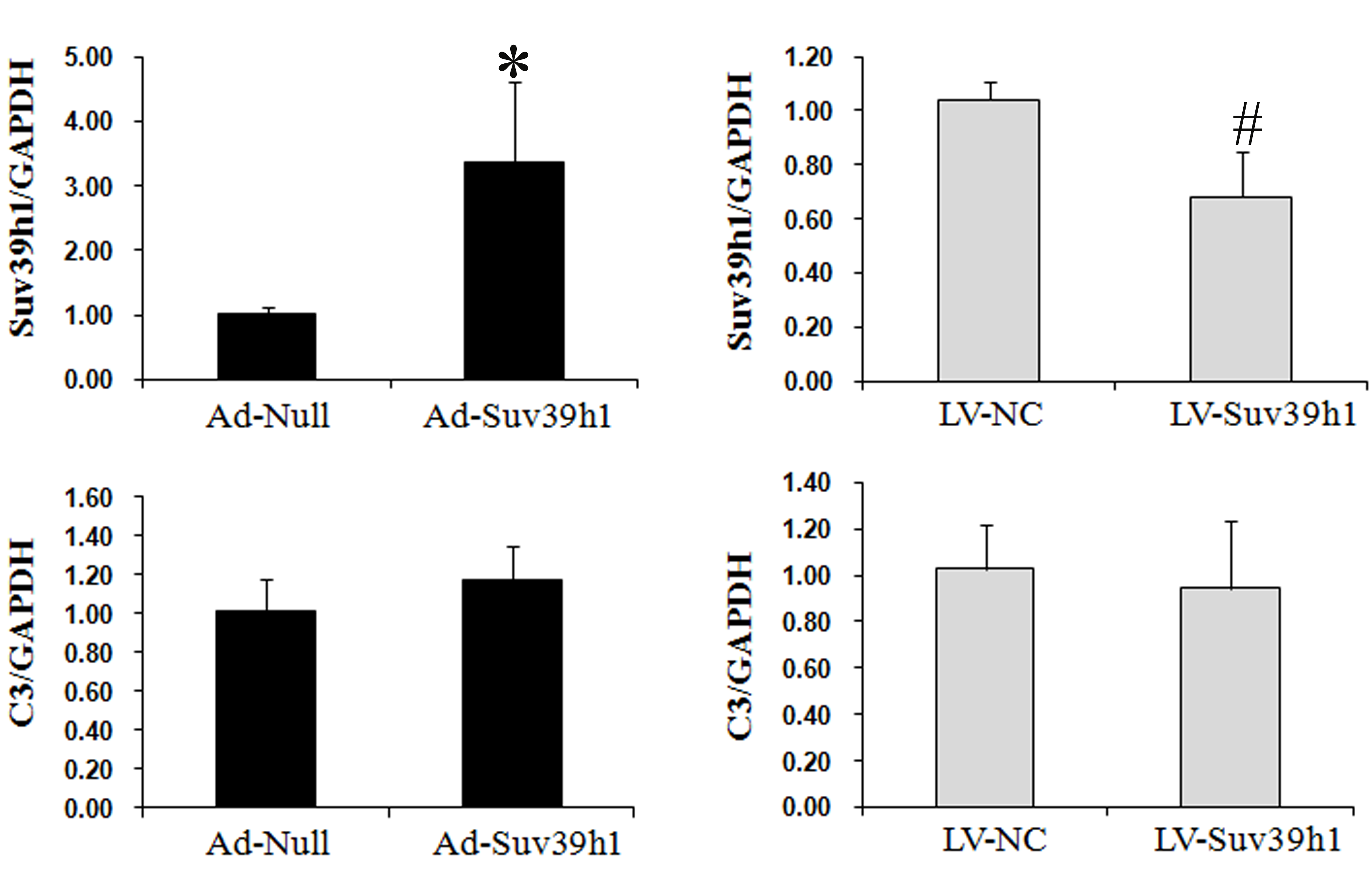

Supplement: Supplementary file 3 [file JCMM-24-973-s003.tif]
