# Supplementary material for: Down‐regulation of Suv39h1 attenuates neointima formation after carotid artery injury in diabetic rats
Source: J Cell Mol Med. 2019 Nov 17;24(1):973–83. doi: 10.1111/jcmm.14809 (PMC6933362; doi:10.1111/jcmm.14809)
Supplement: Supplementary file 4 [file JCMM-24-973-s004.doc]

Table S1. Primer sequences used for quantitative RT-PCR analysis.

| **Target Gene** | **Accession number** | **Forward** | **Reverse** | **bp** |
| --- | --- | --- | --- | --- |
| Suv39h1 | NM_001106956.1 | 5’-CGTGGATGCCGCCTATTATG-3’ | 5’-GGGGTAGTCGCTCATCAAGGT-3’ | 110 |
| Complement C3 | NM_016994.2 | 5’-GCCCAGCCCTACAGCACTAT-3’ | 5’-CCAGGCGGAAGGAAGGTAT-3’ | 268 |
| p15 | NM_130812.3 | 5'-CCACGGAGCAGAACCCAACT- 3' | 5’-CAGCACCATTAGCGTGTCCAG-3’ | 97 |
| p16 | NM_031550.1 | 5’-CTAGAGCGGGGACATCACGA -3’ | 5’- AGTCCTAGGCACCTGGGCG -3’ | 122 |
| GAPDH | NM_017008.4 | 5’-TGGAGAAACCTGCCAAGTATGAT -3’ | 5’-TCAAAGGTGGAAGAATGGGAGT -3’ | 142 |
