# Supplementary material for: Down‐regulation of Suv39h1 attenuates neointima formation after carotid artery injury in diabetic rats
Source: J Cell Mol Med. 2019 Nov 17;24(1):973–83. doi: 10.1111/jcmm.14809 (PMC6933362; doi:10.1111/jcmm.14809)
Supplement: Supplementary file 5 [file JCMM-24-973-s005.docx]

|  | Normal-rat | DM-rat | DM-rat | | | |
| --- | --- | --- | --- | --- | --- | --- |
|  |  |  | Ad-Null | Ad-Suv39h1 | LV-NC | LV-Suv39h1 |
| GLU（mmol/L） | 7.60±0.67 | 24.48±2.30^a^ | 25.93±3.57 | 24.84±9.82 | 25.47±14.81 | 23.96±11.19 |
| TG  （mmol/L） | 1.67±1.41 | 2.07±1.99 | 1.37±0.57 | 1.75±1.58 | 2.86±1.22 | 0.46±0.18^b^ |
| TC  （mmol/L） | 1.48±0.24 | 1.57±0.69 | 1.86±0.37 | 1.65±0.40 | 1.70±0.13 | 1.40±0.58 |
| LDL-C  （mmol/L） | 0.15±0.06 | 0.20±0.19 | 0.18±0.06 | 0.27±0.07 | 0.19±0.08 | 0.18±0.05 |
| BW  (g) | 375.00±20.29 | 345.40±36.62 | 335.40±9.48 | 342±18.87 | 308.00±37.04 | 311.60±24.14 |

Table S2. The blood glucose, insulin, lipids and body weight in animal groups

The blood glucose, lipids and body weight were calculated on day 28 after establishing the carotid artery balloon injury model and infecting the injured vessels with virus vectors. GLU: random blood glucose; TG: triglyceride; TC: total cholesterol; LDL-C: low density lipoprotein cholesterin; BW: body weight. ^a^*P* < 0.05, as compared to the Normal-rat group; ^b^*P* < 0.05, as compared to the LV-NC group (n=5). The results are representative of six separate experiments.
