# Supplementary material for: Down‐regulation of Suv39h1 attenuates neointima formation after carotid artery injury in diabetic rats
Source: J Cell Mol Med. 2019 Nov 17;24(1):973–83. doi: 10.1111/jcmm.14809 (PMC6933362; doi:10.1111/jcmm.14809)
Supplement: Supplementary file 6 [file JCMM-24-973-s006.docx]

**Table S3** Differentially expressed genes between Ad-Null or Ad-Suv39h1 transfected arteries after balloon injury for 7 days in diabetic rats (fold change > 1.5; *p* < 0.05)

| **Gene Symbol** | **Fold Change**  **(Ad-Suv39h1/ Ad-Null)** | **P-value** |
| --- | --- | --- |
| Suv39h1 | 72.35657 | 0.00017 |
| LOC366632 | 3.81432 | 0.021844 |
| Cldn23 | 3.459585 | 0.045762 |
| Clec4e | 3.427701 | 0.016553 |
| Cox7c | 3.424448 | 0.034912 |
| LOC366979 | 3.155215 | 0.002466 |
| S100a9 | 3.149198 | 0.014901 |
| Ly49si1 | 3.063273 | 0.043457 |
| Plp1 | 3.024207 | 0.030372 |
| S100a8 | 3.020581 | 0.035492 |
| RGD1561841 | 3.009756 | 0.045504 |
| Gnal | 2.958317 | 0.04618 |
| LOC498685 | 2.868136 | 0.040057 |
| RGD1306613 | 2.795303 | 0.014771 |
| Krcc1 | 2.48241 | 0.000678 |
| LOC679715 | 2.423671 | 0.015303 |
| Ttll11 | 2.370189 | 0.013099 |
| Rap1gap | 2.360004 | 0.029199 |
| Plcb4 | 2.341273 | 0.042605 |
| Parvg | 2.335151 | 0.012452 |
| LOC684441 | 2.324688 | 0.010312 |
| C3 | 2.307046 | 0.005861 |
| Timd2 | 2.26976 | 0.015514 |
| Il13ra2 | 2.254846 | 0.048906 |
| Scg5 | 2.235305 | 0.043608 |
| Zfp644 | 2.228877 | 0.030858 |
| Ucp3 | 2.219909 | 0.007793 |
| Id3 | 2.217346 | 0.019801 |
| RGD1565030 | 2.184634 | 0.034853 |
| Dynlrb2 | 2.17289 | 0.017889 |
| Colq | 2.163766 | 0.048145 |
| RGD1565622 | 2.151845 | 0.017797 |
| Cpg1 | 2.113715 | 0.01629 |
| Il11 | 2.103713 | 0.025566 |
| LOC690134 | 2.094711 | 0.016622 |
| Gabbr1 | 2.089396 | 0.011019 |
| Rnf208 | 2.084341 | 0.03865 |
| RGD1560058 | 2.07472 | 0.014711 |
| Epha4 | 2.073206 | 5.49E-05 |
| Bdnf | 2.034572 | 0.022828 |
| Nkd2 | 2.016632 | 0.008519 |
| Chm | 2.008369 | 0.034386 |
| Bmp3 | 2.00683 | 0.036456 |
| Matn3 | 2.005375 | 0.000237 |
| Lcn2 | 1.984636 | 0.039361 |
| LOC499843 | 1.952974 | 0.037601 |
| Mrps15 | 1.944243 | 0.000979 |
| Mab21l3 | 1.941611 | 0.035624 |
| Olr1375 | 1.917307 | 0.028461 |
| Mrpl21 | 1.916021 | 0.029826 |
| LOC687071 | 1.899516 | 0.000528 |
| LOC291686 | 1.888724 | 0.026282 |
| Dynlrb2 | 1.887033 | 0.020693 |
| RGD1563613 | 1.886805 | 0.011447 |
| Rgs4 | 1.881353 | 0.025305 |
| B3galt6 | 1.874962 | 0.04823 |
| Eef1a1 | 1.8707 | 0.018451 |
| Hp | 1.866478 | 0.011059 |
| Hsph1 | 1.852656 | 0.040039 |
| Zfp748 | 1.840069 | 0.038143 |
| Scn5a | 1.835557 | 0.019703 |
| Lmcd1 | 1.81904 | 0.033704 |
| Rassf3 | 1.801745 | 0.043627 |
| RGD1564268 | 1.798724 | 0.036956 |
| Lrp4 | 1.783783 | 0.029068 |
| LOC685157 | 1.773778 | 0.016696 |
| Ccdc72 | 1.763412 | 0.016461 |
| Pram1 | 1.760367 | 0.035333 |
| Amn1 | 1.75688 | 0.007649 |
| Fgd3 | 1.756101 | 0.00472 |
| Fbxl22 | 1.753719 | 0.033243 |
| Phldb2 | 1.750377 | 0.015663 |
| Gata6 | 1.75021 | 0.035613 |
| Rab6b | 1.748932 | 0.043664 |
| Ctf1 | 1.747049 | 0.020546 |
| Ndufb4l1 | 1.744646 | 0.029964 |
| Pcyox1l | 1.742908 | 0.049642 |
| RGD1566373 | 1.738276 | 0.043005 |
| Grem1 | 1.738144 | 0.001735 |
| LOC689103 | 1.725796 | 0.021683 |
| Clic2 | 1.7164 | 0.030381 |
| LOC499219 | 1.702161 | 0.007696 |
| Ncam1 | 1.701823 | 0.005888 |
| Gpr182 | 1.694914 | 0.031608 |
| Rsl1d1 | 1.683771 | 0.008191 |
| Ranbp1 | 1.678253 | 0.032993 |
| RGD1564906 | 1.676953 | 0.033955 |
| Cnksr3 | 1.676525 | 0.040953 |
| Chrna1 | 1.673138 | 0.008917 |
| Cck | 1.672166 | 0.020316 |
| Defb24 | 1.663632 | 0.008556 |
| Myh10 | 1.655635 | 0.031015 |
| Ptn | 1.650125 | 0.047219 |
| Tcp11 | 1.643046 | 0.025616 |
| RGD1564463 | 1.640744 | 0.044097 |
| Sgcg | 1.63496 | 0.001618 |
| Nucb2 | 1.634374 | 0.045654 |
| Slc9a9 | 1.633547 | 0.03977 |
| Mip | 1.63333 | 0.039004 |
| Trdmt1 | 1.633005 | 0.03503 |
| Pdlim5 | 1.631738 | 0.018065 |
| Zfp748 | 1.627919 | 0.035021 |
| Cd200 | 1.616307 | 0.00325 |
| Olr472 | 1.613776 | 0.010222 |
| Acsbg1 | 1.611101 | 0.037587 |
| Snai2 | 1.603983 | 0.004816 |
| Scx | 1.603951 | 0.048875 |
| Mcm4 | 1.602952 | 0.043922 |
| RGD1561413 | 1.601647 | 0.008143 |
| Fn3krp | 1.601556 | 0.044037 |
| Olr165 | 1.595229 | 0.045272 |
| Pex19 | 1.584572 | 0.012662 |
| L2hgdh | 1.584002 | 0.011357 |
| LOC690358 | 1.581439 | 0.038794 |
| Gpr21 | 1.575572 | 0.035207 |
| Fbxo5 | 1.570257 | 0.028924 |
| Arsi | 1.566515 | 0.037871 |
| LOC100365604 | 1.562278 | 0.030697 |
| Rpl10l | 1.561083 | 0.037939 |
| B3galnt2 | 1.558579 | 0.012156 |
| Ebf3 | 1.558318 | 0.029308 |
| Fam176a | 1.548955 | 0.04783 |
| Brdt | 1.548896 | 0.01075 |
| Lrrc8e | 1.548035 | 0.0253 |
| Ccl24 | 1.54511 | 0.02399 |
| Bmper | 1.543468 | 0.045444 |
| Nsun6 | 1.537321 | 0.032245 |
| Gpr18 | 1.535949 | 0.023505 |
| Ric8a | 1.534764 | 0.00701 |
| Agtr1a | 1.534548 | 0.004372 |
| Ctxn1 | 1.522029 | 0.01276 |
| Stx11 | 1.52121 | 0.030859 |
| Ftsj1 | 1.51614 | 0.018015 |
| Caprin1 | 1.515041 | 0.033312 |
| Rasl11b | 1.513722 | 0.042008 |
| Dclk1 | 1.512993 | 0.001893 |
| Mitf | 1.50947 | 0.032359 |
| Slc38a6 | 1.50666 | 0.042119 |
| RGD1560648 | 1.505254 | 0.010358 |
| Fgf7 | 1.502388 | 0.007359 |
| Ipo9 | 1.502049 | 0.042943 |
| Ubd | -6.635769 | 0.027029 |
| Cxcl2 | -4.092477 | 0.007125 |
| Cxcl10 | -3.863099 | 0.007019 |
| Cxcl9 | -3.37283 | 0.03099 |
| Ptgs2 | -2.952151 | 0.007603 |
| Nr4a1 | -2.944601 | 0.009249 |
| Gbp5 | -2.854223 | 0.006872 |
| Nkain4 | -2.71172 | 0.022628 |
| Dbp | -2.666456 | 0.001737 |
| LOC686921 | -2.635996 | 0.015513 |
| Cx3cl1 | -2.630257 | 0.007763 |
| Kif13b | -2.605788 | 0.023425 |
| Il10 | -2.574567 | 0.023362 |
| Cd5l | -2.547352 | 0.034673 |
| Dbp | -2.524134 | 0.006177 |
| LOC501302 | -2.513539 | 0.037531 |
| Gbp2 | -2.493331 | 0.005822 |
| Gldn | -2.482888 | 0.009422 |
| RT1-Bb | -2.458132 | 0.032089 |
| Nr1d1 | -2.454537 | 0.003066 |
| Kcnn4 | -2.445117 | 0.00026 |
| Atoh8 | -2.407214 | 0.00226 |
| Slc25a23 | -2.35696 | 0.010891 |
| Klf2 | -2.339455 | 0.013309 |
| Adra1b | -2.322438 | 0.002792 |
| Olr791 | -2.322085 | 0.045009 |
| Prodh | -2.319518 | 0.015114 |
| Lrrn3 | -2.305695 | 0.021828 |
| Fos | -2.305164 | 0.01186 |
| LOC685067 | -2.269435 | 0.013133 |
| Npw | -2.26505 | 0.002451 |
| Dusp1 | -2.216264 | 0.009361 |
| Dbp | -2.151379 | 0.003928 |
| Ccl6 | -2.115277 | 0.01681 |
| Kcnn4 | -2.111697 | 0.00148 |
| Tnxb | -2.070277 | 0.027364 |
| Ifi27l2b | -2.049873 | 0.019175 |
| Cntn2 | -2.036007 | 0.018408 |
| Cxcr4 | -2.03395 | 0.021183 |
| Efemp1 | -2.026487 | 0.049083 |
| Tusc1 | -2.014198 | 0.039624 |
| Hcls1 | -2.003161 | 0.022573 |
| Rad23a | -2.000855 | 0.015049 |
| Igtp | -1.991821 | 0.026941 |
| Pla2g2d | -1.933039 | 0.007616 |
| Gsn | -1.916818 | 0.049852 |
| Lrrn4cl | -1.915509 | 0.003315 |
| Irf1 | -1.914074 | 0.002452 |
| F13a1 | -1.906825 | 0.048762 |
| Asb1 | -1.904685 | 0.028943 |
| Mal | -1.895767 | 0.003076 |
| LOC501110 | -1.892666 | 0.02805 |
| Ptprn | -1.889735 | 0.012453 |
| Ppp1r1a | -1.881104 | 0.021195 |
| Irf1 | -1.876658 | 0.040353 |
| Kcnq1 | -1.872448 | 0.003018 |
| Pgf | -1.8575 | 0.001563 |
| Nptxr | -1.857302 | 0.004955 |
| Tnxa-ps1 | -1.848598 | 0.043072 |
| Gstm7 | -1.845124 | 0.007378 |
| Orai1 | -1.83451 | 0.002962 |
| Ngef | -1.823027 | 0.008994 |
| Atf3 | -1.822684 | 0.009158 |
| Ccl6 | -1.817157 | 0.017034 |
| Btg2 | -1.814077 | 0.00769 |
| Klf4 | -1.801546 | 0.020774 |
| Pcsk1n | -1.785382 | 0.023545 |
| Zfp395 | -1.78133 | 0.007674 |
| C1qc | -1.781187 | 0.045755 |
| Ifi27l2b | -1.769448 | 0.028896 |
| Cbx7 | -1.76402 | 0.045969 |
| Adam15 | -1.76067 | 0.035091 |
| Socs3 | -1.756677 | 0.021599 |
| Itga5 | -1.755021 | 0.001117 |
| Jun | -1.7502 | 0.003572 |
| Ccdc88b | -1.748059 | 0.035751 |
| Inpp5d | -1.744405 | 0.000505 |
| C2 | -1.742587 | 0.03248 |
| Lphn1 | -1.733393 | 0.012131 |
| Mmp24 | -1.733159 | 0.01489 |
| MGC105567 | -1.73257 | 0.047237 |
| Eif2ak1 | -1.726088 | 0.001285 |
| Tpcn1 | -1.724292 | 0.010085 |
| Lrrk1 | -1.722209 | 0.029308 |
| MGC108823 | -1.721131 | 0.027443 |
| Mycbpap | -1.720584 | 0.005769 |
| Kcnk3 | -1.719247 | 0.01445 |
| C2 | -1.715989 | 0.048699 |
| Slc9a3r2 | -1.709434 | 0.003341 |
| Twist2 | -1.706398 | 0.002161 |
| Tgfb1 | -1.703775 | 0.01137 |
| Heyl | -1.70315 | 0.015215 |
| Smug1 | -1.698422 | 0.028703 |
| Has1 | -1.692504 | 0.027582 |
| Gtpbp6 | -1.691949 | 0.000587 |
| Plekhg5 | -1.691383 | 0.00419 |
| Lst1 | -1.687126 | 0.001896 |
| Lrfn3 | -1.671815 | 0.008264 |
| LOC301748 | -1.669542 | 0.033555 |
| LOC314328 | -1.667014 | 0.023893 |
| Smcr7 | -1.660358 | 0.013075 |
| Slc6a2 | -1.660013 | 0.043089 |
| LOC681825 | -1.657179 | 0.022675 |
| Gp1bb | -1.655806 | 0.007583 |
| C1qa | -1.648138 | 0.02408 |
| Fxyd2 | -1.635565 | 0.013517 |
| Rimklb | -1.632886 | 0.013443 |
| Acer2 | -1.632274 | 0.041299 |
| Tst | -1.629033 | 0.002864 |
| Lrrc3b | -1.625343 | 0.002251 |
| Atp1a2 | -1.624476 | 0.003925 |
| Slc24a6 | -1.621957 | 0.00834 |
| Cd180 | -1.621951 | 0.044675 |
| Fez1 | -1.616968 | 0.031817 |
| Fbxo46 | -1.610487 | 0.02484 |
| Ahnak | -1.605192 | 0.01067 |
| Ppp1r15a | -1.604723 | 0.00223 |
| Tlx3 | -1.602972 | 0.040408 |
| Dmwd | -1.599764 | 0.025618 |
| Plin3 | -1.598844 | 0.041735 |
| Slc7a5 | -1.596775 | 0.005231 |
| Ace | -1.593706 | 0.022392 |
| Mri1 | -1.5929 | 0.044255 |
| Kcnk5 | -1.589312 | 0.020049 |
| Tpd52l1 | -1.588443 | 0.013256 |
| Phlda3 | -1.585298 | 0.028723 |
| Rasl10a | -1.582885 | 0.025318 |
| Flad1 | -1.582514 | 0.020299 |
| Plekhg2 | -1.580867 | 0.011152 |
| Hist2h3c2 | -1.579754 | 0.021728 |
| RGD1309362 | -1.579544 | 0.029849 |
| Fbxo6 | -1.575097 | 0.022901 |
| Rnf187 | -1.57431 | 0.029194 |
| Usp18 | -1.572971 | 0.013022 |
| Atp1b2 | -1.571998 | 0.003776 |
| Caskin2 | -1.571702 | 0.020929 |
| Psmb9 | -1.568797 | 0.047181 |
| LOC691777 | -1.568439 | 0.048144 |
| Pcbp4 | -1.568327 | 0.012587 |
| Gstm4 | -1.566757 | 0.006329 |
| Ntf3 | -1.566331 | 0.046493 |
| Wdr91 | -1.564856 | 0.025971 |
| Nr4a2 | -1.563636 | 0.005902 |
| Nes | -1.56185 | 0.000125 |
| Taok2 | -1.561021 | 0.009331 |
| Col27a1 | -1.560565 | 0.009131 |
| Amdhd2 | -1.558501 | 0.001456 |
| Pde4a | -1.558263 | 0.036212 |
| Olfm2 | -1.557388 | 0.037187 |
| Slc16a8 | -1.556769 | 0.014903 |
| Xcl1 | -1.553962 | 0.048367 |
| Plaur | -1.553115 | 0.004405 |
| Mybl2 | -1.551877 | 0.007478 |
| Cldn22 | -1.550542 | 0.026982 |
| Fgf18 | -1.548648 | 0.027494 |
| Car3 | -1.548291 | 0.006057 |
| Junb | -1.548104 | 0.034332 |
| Pacrg | -1.547734 | 0.042107 |
| Tmem121 | -1.546735 | 0.000942 |
| RGD1305793 | -1.54484 | 0.004261 |
| Epn1 | -1.542635 | 0.018709 |
| Spi1 | -1.541146 | 0.030485 |
| Usp18 | -1.540277 | 0.01582 |
| Nbl1 | -1.531977 | 0.01828 |
| Epn2 | -1.530161 | 0.028518 |
| Slamf8 | -1.529867 | 0.005282 |
| Tob2 | -1.528529 | 0.021382 |
| Trim41 | -1.527076 | 0.013517 |
| Egln2 | -1.526466 | 0.020859 |
| Scube1 | -1.525977 | 0.042501 |
| Phactr1 | -1.525049 | 0.000823 |
| Plk3 | -1.521549 | 0.007478 |
| Ppp1r16a | -1.520556 | 0.029607 |
| Gprc5b | -1.518696 | 0.039672 |
| Hsd17b8 | -1.516041 | 0.000142 |
| RGD1307569 | -1.514901 | 0.025887 |
| Tnfrsf21 | -1.511687 | 0.007939 |
| Tmem121 | -1.511504 | 0.002162 |
| Tet2 | -1.509286 | 0.031517 |
| Larp6 | -1.50831 | 0.038457 |
| Rbm3 | -1.507991 | 0.021663 |
| Por | -1.505885 | 0.038972 |
| Pycrl | -1.505678 | 0.014296 |
| S100a1 | -1.505364 | 0.03108 |
| Ahnak | -1.503261 | 0.003563 |
