# Supplementary material for: Down‐regulation of Suv39h1 attenuates neointima formation after carotid artery injury in diabetic rats
Source: J Cell Mol Med. 2019 Nov 17;24(1):973–83. doi: 10.1111/jcmm.14809 (PMC6933362; doi:10.1111/jcmm.14809)
Supplement: Supplementary file 7 [file JCMM-24-973-s007.docx]

**Table S4** Differentially expressed genes between LV-NC or LV-Suv39h1-transfected arteries after balloon injury for 7 days in diabetic rats (fold change > 1.5; *p* < 0.05).

| **Gene Symbol** | **Fold Change** | **P-value** |
| --- | --- | --- |
|  | **(LV-NC / LV-Suv39h1)** |  |
| Vsig4 | 10.88462325 | 0.0489101 |
| Sult1b1 | 10.39417417 | 0.03842553 |
| Rnf6 | 9.813735806 | 0.00218519 |
| Sult1b1 | 8.350829637 | 0.02448191 |
| Rbbp4 | 7.891832668 | 0.00097364 |
| Bmp6 | 6.420623636 | 0.02595637 |
| Cytl1 | 6.283254426 | 3.09E-05 |
| Zfp641 | 5.538814671 | 0.00123985 |
| Ccl20 | 5.338647646 | 0.00078495 |
| RGD1309291 | 5.259073768 | 0.0008507 |
| Ddo | 4.726162169 | 0.0441885 |
| Olr1555 | 4.526923416 | 0.01631876 |
| Vom1r28 | 4.391189313 | 0.01774603 |
| Slamf6 | 4.36562164 | 0.02083816 |
| LOC100363193 | 4.211021087 | 0.0232932 |
| Ptx3 | 4.209606263 | 0.00373854 |
| LOC362795 | 4.077574304 | 0.02572038 |
| Slc7a12 | 3.893277557 | 0.0040874 |
| Ptgs2 | 3.85518876 | 2.90E-05 |
| Cytl1 | 3.781778587 | 0.00017999 |
| RGD1560455 | 3.744847839 | 0.04763496 |
| Testin | 3.728268165 | 0.04072552 |
| Pkhd1l1 | 3.718868926 | 0.0117131 |
| Grpca | 3.695800376 | 0.03348553 |
| Vsig8 | 3.694143135 | 0.00191562 |
| Adamts19 | 3.677733904 | 4.16E-05 |
| Bmx | 3.486808182 | 0.00167029 |
| Uhrf1 | 3.474050599 | 0.0122593 |
| LOC683891 | 3.473475208 | 0.00682138 |
| Ctnnb1 | 3.393939388 | 0.00113785 |
| Cpne6 | 3.386143624 | 0.0235706 |
| LOC100361882 | 3.373505779 | 0.01771221 |
| Tmtc2 | 3.369914293 | 0.03354118 |
| Hist3h2bb | 3.269097185 | 0.00775605 |
| Olr25 | 3.264279066 | 0.0395832 |
| LOC312273 | 3.192560442 | 0.01561313 |
| Ccdc68 | 3.189327905 | 0.00034069 |
| Pnoc | 3.164361008 | 0.01622109 |
| Hsd3b7 | 3.138034253 | 0.00135636 |
| Gpr119 | 3.109885577 | 0.0370632 |
| Gnat2 | 3.10162478 | 5.63E-05 |
| LOC499806 | 3.093656604 | 0.03137375 |
| Olr952 | 3.09243749 | 0.01828539 |
| RGD1563400 | 3.085948174 | 0.0064164 |
| Cxcl5 | 3.082033408 | 0.00054288 |
| Lctl | 3.077268938 | 0.00478533 |
| Ptx3 | 3.044568065 | 0.01292935 |
| Fmo4 | 3.041652067 | 0.01412431 |
| Scel | 3.031536095 | 0.01322406 |
| Ythdf2 | 3.021620618 | 2.23E-05 |
| Pcnxl2 | 3.01579612 | 0.00781063 |
| Fmo2 | 2.963835068 | 0.00568003 |
| Bmp8a | 2.962678271 | 0.00091962 |
| Lpar4 | 2.956311996 | 0.04781975 |
| Rsu1 | 2.950043715 | 0.02486414 |
| Ptgds | 2.943370321 | 0.04840551 |
| Gal | 2.907480602 | 0.00282624 |
| LOC685408 | 2.875972738 | 0.0020471 |
| LOC687964 | 2.865299804 | 0.04176979 |
| Lsr | 2.834556907 | 0.02920117 |
| Lsr | 2.824957115 | 0.04854986 |
| Pigg | 2.822845169 | 0.03675922 |
| Jdp2 | 2.812306496 | 0.02688832 |
| Slc7a11 | 2.810199072 | 0.00884618 |
| Lrrc4 | 2.807860705 | 0.01300227 |
| Ccr2 | 2.799018133 | 3.95E-05 |
| Olr259 | 2.788470935 | 0.01276597 |
| Ppyr1 | 2.753800029 | 0.04972028 |
| Lmod1 | 2.748333664 | 0.00668069 |
| Kcng1 | 2.729871546 | 0.00323896 |
| Sgk3 | 2.71293986 | 0.00759429 |
| Dync2h1 | 2.70607758 | 0.00636428 |
| Cyp39a1 | 2.70603275 | 0.00269279 |
| Lao1 | 2.690373922 | 3.56E-05 |
| Fmo3 | 2.675447402 | 0.03387413 |
| Dnah2 | 2.671891764 | 0.04746539 |
| Ccl20 | 2.671703606 | 0.00234776 |
| Pllp | 2.668348249 | 0.03938622 |
| RGD1565374 | 2.665144582 | 0.00018611 |
| Mmp9 | 2.637503424 | 0.01201598 |
| Cpne6 | 2.626551732 | 0.01501746 |
| Mcoln2 | 2.603618313 | 0.00108754 |
| Gda | 2.596599183 | 0.01029858 |
| Zar1l | 2.586011696 | 0.0212216 |
| LOC365778 | 2.583406554 | 0.01564879 |
| Gna14 | 2.581204359 | 0.00153645 |
| RGD1305939 | 2.575217823 | 0.00110098 |
| Fmo1 | 2.573588391 | 0.01158498 |
| Cyp1a2 | 2.568098788 | 0.02286572 |
| RGD1563046 | 2.561802281 | 0.03578033 |
| Kcnj1 | 2.557543302 | 0.04785534 |
| RGD1559677 | 2.548509975 | 0.00302612 |
| RT1-Ba | 2.544823618 | 0.03959066 |
| F2r | 2.537838036 | 0.0156961 |
| Zc3h6 | 2.532205341 | 0.01112656 |
| Abca5 | 2.531809752 | 0.02995305 |
| Prg4 | 2.522556063 | 0.02729457 |
| Atad2 | 2.521445775 | 0.03226912 |
| Ibsp | 2.519103387 | 5.69E-05 |
| Tspan11 | 2.506987321 | 0.04093266 |
| Il25 | 2.50221045 | 0.04655004 |
| RGD1308160 | 2.492423573 | 0.035743 |
| LOC100361198 | 2.486894317 | 0.01541888 |
| Msr1 | 2.472812011 | 0.00730769 |
| Cd84 | 2.468470493 | 0.00785692 |
| Cftr | 2.457262982 | 0.03303922 |
| Lypd1 | 2.454867061 | 0.01191568 |
| Ryr2 | 2.452172607 | 0.04944407 |
| Tmem211 | 2.448992123 | 0.04121663 |
| Nrsn1 | 2.447568494 | 0.01469158 |
| Trh | 2.432530173 | 0.00340718 |
| Ccdc64 | 2.431725979 | 0.00706108 |
| Slc15a2 | 2.423196837 | 0.02929108 |
| LOC502684 | 2.416099943 | 0.00183812 |
| Ccdc121 | 2.410507803 | 0.04830655 |
| Fmo2 | 2.409223662 | 0.04474448 |
| Gal | 2.407452778 | 0.00236504 |
| Prpf31 | 2.400138402 | 0.04925016 |
| LOC689416 | 2.397638298 | 0.00020565 |
| Slc6a2 | 2.390077059 | 0.02929539 |
| RGD1562515 | 2.380914808 | 0.02966982 |
| LOC503192 | 2.36878419 | 0.02234031 |
| Nf1 | 2.349629906 | 0.02490887 |
| LOC305806 | 2.348790492 | 0.00980558 |
| F5 | 2.343037753 | 0.0207927 |
| Krtap3-1 | 2.337289868 | 0.04722848 |
| Il17a | 2.329204536 | 0.02873891 |
| Cyp4f18 | 2.315810921 | 0.00024462 |
| Zfyve28 | 2.311842371 | 0.03819146 |
| Uxs1 | 2.31061661 | 0.00919414 |
| RGD1563667 | 2.307984497 | 0.03335535 |
| Ccl17 | 2.304397131 | 0.03996611 |
| LOC688874 | 2.298158126 | 0.03867373 |
| Tert | 2.298127807 | 0.03970234 |
| Fam163a | 2.297965279 | 0.04359855 |
| LOC681227 | 2.293083887 | 0.01906878 |
| RGD1560927 | 2.291525436 | 0.03123187 |
| Nr1h5 | 2.290429251 | 0.04109752 |
| Gpatc2 | 2.287635132 | 0.03040738 |
| Camsap1l1 | 2.28676223 | 0.00021424 |
| Abcg4 | 2.275367641 | 0.01204925 |
| LOC689756 | 2.274453595 | 0.00120467 |
| A4galt | 2.273273552 | 0.01713747 |
| Snx29 | 2.271427881 | 0.03915226 |
| RGD1560455 | 2.27010464 | 0.01067749 |
| RT1-Bb | 2.263148104 | 0.01832648 |
| Vom1r40 | 2.257907681 | 0.04667652 |
| Gtf2a1l | 2.256675948 | 0.0058878 |
| LOC681198 | 2.247828913 | 0.00487672 |
| RGD1560978 | 2.246931277 | 0.00308132 |
| LOC680029 | 2.239936033 | 0.00441703 |
| Pcdh20 | 2.237634392 | 0.00568837 |
| Ppap2b | 2.237198343 | 0.02895686 |
| Fmn1 | 2.235809547 | 0.01333127 |
| Zik1 | 2.233263526 | 0.04725149 |
| Myo5b | 2.228123139 | 0.03639643 |
| Rpesp | 2.225997028 | 0.01018966 |
| Ncf2 | 2.221889033 | 0.03785434 |
| LOC24906 | 2.218583471 | 0.00017865 |
| Gjb6 | 2.216788861 | 0.02742109 |
| Kmo | 2.216160958 | 0.00036435 |
| Usp53 | 2.212226851 | 0.04238074 |
| RGD1565989 | 2.194700993 | 0.00956035 |
| Mettl11b | 2.191843056 | 0.03241262 |
| Atp7a | 2.190627623 | 0.03484356 |
| Fam172a | 2.186235476 | 0.04754742 |
| Tm4sf19 | 2.185871057 | 0.03956937 |
| Fgf14 | 2.185676826 | 0.02584455 |
| Bai3 | 2.183105876 | 0.04148383 |
| Col9a1 | 2.181705493 | 0.01855621 |
| LOC689919 | 2.178534811 | 0.02499844 |
| RGD1563556 | 2.169843362 | 0.00313826 |
| Adamts5 | 2.166515012 | 0.01369514 |
| Vcsa1 | 2.165349445 | 0.01700087 |
| Pi16 | 2.163796265 | 0.03909041 |
| L1cam | 2.154777342 | 0.04899662 |
| Tmem47 | 2.152235677 | 0.03606825 |
| Cwf19l1 | 2.146064743 | 0.04089096 |
| Clec4e | 2.143021068 | 0.01190153 |
| Nap1l2 | 2.141772384 | 0.00428698 |
| Flrt3 | 2.140025815 | 0.00876823 |
| Areg | 2.138077626 | 0.02425838 |
| Poli | 2.131876552 | 0.01112392 |
| Nlgn1 | 2.126696065 | 0.04322616 |
| Tsc22d2 | 2.122805316 | 0.00395848 |
| RGD1565779 | 2.10527087 | 0.04707947 |
| Oprk1 | 2.103703136 | 0.03003315 |
| Bco2 | 2.103422845 | 0.01137016 |
| Tmem67 | 2.102419944 | 0.02825122 |
| Nell2 | 2.100281008 | 0.01161827 |
| Naip5 | 2.099049953 | 0.04755054 |
| Olr1424 | 2.098365171 | 0.00282899 |
| Scel | 2.097922766 | 0.02183599 |
| Slc44a1 | 2.096825494 | 0.00960531 |
| Zfp644 | 2.095344607 | 0.0085646 |
| Zfp418 | 2.090360691 | 0.03198393 |
| Zfp286a | 2.0862547 | 0.00466276 |
| Arhgef9 | 2.07928291 | 0.04084684 |
| RGD1559714 | 2.075521821 | 0.02884174 |
| RGD1307365 | 2.074788579 | 0.00427239 |
| Atp7a | 2.074372473 | 0.03704633 |
| Crhr2 | 2.070046104 | 0.04654221 |
| LOC363060 | 2.068449927 | 0.00772869 |
| Pcdh11x | 2.065296528 | 0.03981885 |
| Ccdc154 | 2.056966598 | 0.00103138 |
| Aldh3a1 | 2.056116723 | 0.02258241 |
| LOC100125385 | 2.052614119 | 0.02418806 |
| Ntm | 2.052579356 | 0.00484465 |
| Nppb | 2.052083639 | 0.00088336 |
| Zfp192 | 2.051997728 | 0.03597185 |
| Pogz | 2.051273697 | 0.00679851 |
| Gna14 | 2.04913069 | 0.00078604 |
| Dmp1 | 2.046362407 | 0.03687985 |
| C1ql3 | 2.045863654 | 0.00234214 |
| Fgf7 | 2.044051813 | 0.04341385 |
| Phactr2 | 2.043005423 | 0.04322746 |
| RGD1562877 | 2.042806187 | 0.00419358 |
| Malt1 | 2.042518577 | 0.02827021 |
| Etv1 | 2.041974854 | 0.01283928 |
| RGD1308023 | 2.041201726 | 0.037843 |
| Scrg1 | 2.041001345 | 0.01222575 |
| LOC300308 | 2.039457203 | 0.00051145 |
| Gpc3 | 2.037412827 | 0.01937724 |
| Mmp12 | 2.035398575 | 0.04896667 |
| Cabp7 | 2.033759568 | 0.04559779 |
| Brdt | 2.032425683 | 0.01018625 |
| Alox12b | 2.032188601 | 0.04337188 |
| Ccl3 | 2.024275054 | 0.00149473 |
| Cyp2c13 | 2.017603523 | 0.00434302 |
| Vom2r34 | 2.015705678 | 0.00106805 |
| Angptl4 | 2.013424907 | 0.00031977 |
| Ephx4 | 2.011066842 | 0.00618655 |
| Grip1 | 2.007453032 | 0.01944046 |
| Trem3 | 2.006534553 | 0.02677718 |
| Gria4 | 2.006081381 | 0.0236216 |
| Zc3h6 | 2.005897008 | 0.0003014 |
| Nfxl1 | 1.998764316 | 0.02416528 |
| Tcf21 | 1.997237823 | 0.01690296 |
| Duox1 | 1.996681195 | 0.00714286 |
| Hmmr | 1.9939799 | 0.0014881 |
| Mpzl2 | 1.990726693 | 0.04596195 |
| Acbd7 | 1.989397681 | 0.00196215 |
| Rhobtb3 | 1.988509103 | 0.00650336 |
| Diaph2 | 1.986883707 | 0.02195681 |
| Slc24a5 | 1.98235462 | 0.0300666 |
| Lrrn4cl | 1.977499505 | 0.01931373 |
| LOC498155 | 1.974115864 | 0.00415202 |
| Oit3 | 1.97371931 | 0.01736817 |
| Bdnf | 1.973714339 | 0.00209124 |
| Snx15 | 1.971670446 | 0.02144273 |
| Cacna2d2 | 1.963935796 | 0.02702536 |
| RGD1562024 | 1.963586473 | 0.0391429 |
| Aebp2 | 1.961866038 | 0.02802635 |
| Slc6a6 | 1.961439269 | 0.00497213 |
| Tex14 | 1.955510433 | 0.01824257 |
| Ccdc39 | 1.955197754 | 0.00067876 |
| Ccl9 | 1.954346804 | 0.02299844 |
| Spry4 | 1.953341912 | 0.02992181 |
| Aass | 1.952209343 | 0.01373089 |
| Lyve1 | 1.951238325 | 0.04067777 |
| LOC292199 | 1.948393173 | 0.00955116 |
| Crygd | 1.945782472 | 0.04306099 |
| Acer2 | 1.943199692 | 0.0138095 |
| Cxcl1 | 1.941651213 | 0.04440238 |
| LOC290595 | 1.940187704 | 0.0005124 |
| Gpr39 | 1.939709988 | 0.02691096 |
| Serinc2 | 1.939671715 | 0.0446328 |
| Mapk4 | 1.937114457 | 0.00057584 |
| Zfp569 | 1.934041296 | 0.00928176 |
| Mpzl2 | 1.932924249 | 0.04882969 |
| Dync1li1 | 1.932316567 | 0.00016973 |
| Nlrp3 | 1.931899439 | 0.00953094 |
| LOC689207 | 1.929361523 | 0.04205957 |
| Lhx8 | 1.920683983 | 3.42E-05 |
| Tgm1 | 1.920301933 | 0.01555706 |
| Taf7 | 1.919468077 | 0.02454397 |
| Adamts1 | 1.917893132 | 0.00576762 |
| Htr2b | 1.917847712 | 0.00596807 |
| Cldn1 | 1.91775559 | 0.03916415 |
| RGD1305347 | 1.91369529 | 0.03048706 |
| Tmem204 | 1.913326389 | 0.00650549 |
| Ptgfr | 1.91304039 | 0.00066253 |
| Msln | 1.91245924 | 0.00230983 |
| Il1rn | 1.910320892 | 0.00269869 |
| LOC294497 | 1.908038568 | 0.00725615 |
| LOC687736 | 1.907555809 | 0.00480176 |
| Lgi3 | 1.905995967 | 0.04575897 |
| Spink8 | 1.903011131 | 0.03480893 |
| LOC691522 | 1.899682829 | 0.00496367 |
| Irf6 | 1.896982145 | 0.00533518 |
| Prpf38b | 1.891591635 | 0.01192022 |
| Fam118a | 1.888241073 | 0.03530932 |
| Il7r | 1.878943997 | 0.00181429 |
| Hsd17b3 | 1.877783236 | 0.00481311 |
| Efcab1 | 1.873581151 | 0.02139583 |
| N4bp2l1 | 1.872895493 | 0.02216618 |
| Dsp | 1.871546422 | 0.00761239 |
| Olr855 | 1.869094311 | 0.00850287 |
| Kng1 | 1.867719626 | 0.04490145 |
| Snai2 | 1.867126532 | 0.00011009 |
| Clec7a | 1.863904401 | 0.00013995 |
| Epb41l3 | 1.862213949 | 0.0113342 |
| Rassf9 | 1.860372088 | 0.02634851 |
| Sycp2 | 1.858603419 | 0.03629716 |
| LOC689410 | 1.858375578 | 0.01703514 |
| Nox4 | 1.857784851 | 0.02413903 |
| Nat2 | 1.857045847 | 0.009691 |
| Rgs4 | 1.855257792 | 0.00067202 |
| Setdb1 | 1.853966146 | 0.00072272 |
| Rcbtb1 | 1.851820163 | 0.01377593 |
| LOC100361581 | 1.850837327 | 0.03213323 |
| Pdk4 | 1.850513508 | 0.02342855 |
| Mmrn1 | 1.850317696 | 0.0004824 |
| Ttc14 | 1.849597859 | 0.04539729 |
| Pvrl4 | 1.848671601 | 0.0002229 |
| Mthfd2 | 1.847106434 | 0.04739466 |
| Lrrc7 | 1.845913863 | 0.00425274 |
| Zbtb41 | 1.845846222 | 0.01478681 |
| Tppp3 | 1.84499448 | 0.04154423 |
| Cyp11a1 | 1.84172386 | 0.00266763 |
| LOC685046 | 1.840707341 | 0.00605936 |
| MAST1 | 1.839691637 | 0.0409133 |
| RGD1359334 | 1.838581845 | 0.00944343 |
| LOC100363994 | 1.838535755 | 0.03394458 |
| Isl1 | 1.838336453 | 0.02095594 |
| Casc1 | 1.835746268 | 0.0011517 |
| Cirbp | 1.834390938 | 0.00032909 |
| RGD1565059 | 1.832267064 | 0.01767482 |
| Pid1 | 1.831485992 | 0.00440602 |
| Dlg2 | 1.831205668 | 0.01605866 |
| LOC685904 | 1.830817812 | 0.00065802 |
| Abca16 | 1.828585942 | 0.00723936 |
| Naa11 | 1.825868544 | 0.00226599 |
| Car13 | 1.824677543 | 0.0034106 |
| Zfp141 | 1.823660698 | 0.00226359 |
| Usp53 | 1.821781202 | 0.02707638 |
| Zfp334 | 1.821215235 | 0.00239629 |
| Pgr15l | 1.819152009 | 0.02085482 |
| Grem2 | 1.81795619 | 0.00136935 |
| Spag8 | 1.816674227 | 0.00794341 |
| Ar | 1.815378865 | 0.0412484 |
| Hs3st2 | 1.814497162 | 0.03664285 |
| Gcnt1 | 1.813943014 | 0.00406153 |
| LOC681908 | 1.813273861 | 0.03339728 |
| Meis2 | 1.81289328 | 0.04966896 |
| Ly49i3 | 1.811972173 | 4.19E-05 |
| Cwf19l2 | 1.811279391 | 0.03677505 |
| Zfp667 | 1.802997046 | 0.01695516 |
| Olr799 | 1.802971135 | 0.01570178 |
| Lmln | 1.799810357 | 0.04008523 |
| Ghrhr | 1.795671897 | 0.04522615 |
| LOC688635 | 1.795609126 | 0.00220505 |
| Gpr84 | 1.794139 | 0.01265022 |
| Ccdc37 | 1.789838397 | 0.01762233 |
| Sesn1 | 1.789251886 | 0.01014128 |
| Has2 | 1.788488366 | 0.00306608 |
| Cd47 | 1.785930163 | 0.00998628 |
| Atp6v1g2 | 1.785398394 | 0.01185064 |
| Otud7a | 1.784153941 | 0.0155657 |
| Pcdhb21 | 1.783051822 | 0.04773678 |
| Tmem100 | 1.778814208 | 0.00686514 |
| Rab38 | 1.777868764 | 0.00069608 |
| Eml5 | 1.773184185 | 0.03840178 |
| Pla2g7 | 1.772841428 | 0.0322636 |
| Map2k6 | 1.765564589 | 0.01696046 |
| Tomm20l | 1.765439032 | 0.02406592 |
| Lonrf1 | 1.764560098 | 0.00473996 |
| Tnf | 1.758907808 | 0.0027321 |
| Pcdh8 | 1.757718979 | 0.00744082 |
| Gigyf1 | 1.757113925 | 0.01641113 |
| Itgb2 | 1.756501327 | 0.00269046 |
| Epb41l3 | 1.754941492 | 0.03366667 |
| RGD1309110 | 1.752500635 | 0.03374631 |
| LOC501110 | 1.751944817 | 0.01875782 |
| Plscr4 | 1.751782506 | 0.01364727 |
| Arsk | 1.751087651 | 0.04141708 |
| Hist3h2a | 1.750819066 | 0.00619461 |
| LOC683514 | 1.750158844 | 0.00086641 |
| Lat2 | 1.749733011 | 0.01211786 |
| Akr1c18 | 1.748899879 | 0.03196292 |
| Nim1 | 1.748839914 | 0.01322033 |
| Selenbp1 | 1.748824479 | 0.00190623 |
| Atp8b4 | 1.747434323 | 0.04431488 |
| Filip1l | 1.74689997 | 0.004365 |
| Ddhd2 | 1.746728965 | 0.01299448 |
| Helb | 1.743442439 | 0.00053703 |
| Masp2 | 1.742606263 | 0.00783262 |
| Prrt1 | 1.742148697 | 0.02181142 |
| RGD1561465 | 1.739739299 | 0.0400406 |
| Lilrc2 | 1.738815185 | 0.026336 |
| Rufy2 | 1.737797003 | 0.00906917 |
| Lig4 | 1.737132942 | 0.01723556 |
| LOC688399 | 1.736087024 | 0.0483736 |
| Krt28 | 1.735911502 | 0.03087882 |
| Sema3e | 1.735707725 | 0.00060629 |
| Gpr101 | 1.734887043 | 0.0390175 |
| Epyc | 1.731192315 | 0.02673909 |
| Lrriq3 | 1.730902106 | 0.00730291 |
| Fyb | 1.730068467 | 0.00080044 |
| Fut7 | 1.728095802 | 0.02636663 |
| RGD1564712 | 1.727508489 | 0.03727353 |
| Nasp | 1.726602082 | 0.01862753 |
| Itga4 | 1.724656641 | 0.00178598 |
| Akr1e2 | 1.723373613 | 0.00039078 |
| Hs3st1 | 1.720569497 | 0.00032491 |
| Zfp36l1 | 1.719445196 | 0.01124026 |
| LOC691797 | 1.719118427 | 0.00585156 |
| Pabpc4l | 1.716369695 | 0.02590339 |
| Arhgef5 | 1.715833582 | 0.00451269 |
| Olr1229 | 1.714956045 | 0.02766817 |
| Map7d2 | 1.713494705 | 0.01662363 |
| Sntg2 | 1.713442842 | 0.03073792 |
| Lilrb4 | 1.710224438 | 0.04608536 |
| Fam26e | 1.708596422 | 0.00022167 |
| Cage1 | 1.705983508 | 0.01475257 |
| Nfat5 | 1.705934829 | 0.00450652 |
| Lum | 1.705354102 | 2.53E-05 |
| Dnajb4 | 1.704344133 | 0.02028143 |
| Akr1c19 | 1.703726393 | 0.04814519 |
| Clec5a | 1.702546262 | 0.02577946 |
| Kdm4d | 1.701270839 | 0.00507482 |
| Arhgef5 | 1.697121084 | 0.00699041 |
| Sparcl1 | 1.69650263 | 0.00614715 |
| Fbxo3 | 1.696358664 | 0.016855 |
| LOC100360592 | 1.695882991 | 0.03757669 |
| Alpk1 | 1.694353381 | 0.01217344 |
| Acer2 | 1.694319166 | 0.03720702 |
| Grem2 | 1.694105828 | 0.00185927 |
| LOC296884 | 1.693883358 | 0.04680862 |
| Trim13 | 1.693774561 | 0.02932481 |
| Cat | 1.69355597 | 0.04789583 |
| Shprh | 1.691482206 | 0.02956274 |
| Rapgef5 | 1.690497637 | 0.00432657 |
| Duox2 | 1.690007521 | 2.93E-05 |
| Fap | 1.690000492 | 0.01799733 |
| Vom2r45 | 1.689942235 | 0.0364012 |
| Olr1587 | 1.689797146 | 0.02221036 |
| Slfn1 | 1.689634229 | 0.02468069 |
| RGD1305939 | 1.68773126 | 0.02539675 |
| LOC685792 | 1.686351367 | 0.02139186 |
| Anapc1 | 1.685043692 | 2.71E-05 |
| Bhlhb9 | 1.684495958 | 0.02404299 |
| Dhx40 | 1.684124351 | 0.03311019 |
| Lyst | 1.68387483 | 0.03865898 |
| Satb2 | 1.68167121 | 0.01619416 |
| Pwwp2a | 1.680441477 | 0.03335496 |
| RGD1560203 | 1.679696601 | 0.01730654 |
| Uba6 | 1.678959426 | 0.03544576 |
| Vom2r47 | 1.678897787 | 0.01436679 |
| Slc25a40 | 1.675377689 | 0.03405629 |
| Il10ra | 1.673034428 | 0.00569134 |
| Pcbd1 | 1.672451066 | 0.04066662 |
| Rnf39 | 1.672096372 | 0.03976134 |
| Cxcr7 | 1.671888536 | 0.00341472 |
| Rnd3 | 1.670905142 | 0.04155831 |
| Asah1 | 1.670380953 | 0.01653567 |
| Paip1 | 1.669288907 | 0.01646308 |
| Ptger4 | 1.668267803 | 0.01680198 |
| Edn1 | 1.667565003 | 0.01002272 |
| Plekhh1 | 1.664914549 | 0.0209937 |
| RGD1563349 | 1.663660782 | 0.0420535 |
| Akr1e2 | 1.662938291 | 0.00179532 |
| Lcn2 | 1.662415488 | 0.02056389 |
| Slc4a7 | 1.661758039 | 0.00799648 |
| LOC500013 | 1.661526919 | 0.04367309 |
| Pdgfd | 1.66059098 | 0.01843626 |
| Dph1 | 1.660387566 | 0.04002363 |
| Fscn2 | 1.660294807 | 0.01856061 |
| Pdgfra | 1.659582098 | 0.00107192 |
| St8sia4 | 1.658176218 | 0.0098026 |
| Insig2 | 1.657981604 | 0.01512693 |
| Ptgs1 | 1.657895415 | 0.00636981 |
| Ing3 | 1.656370982 | 0.00370825 |
| Col17a1 | 1.655061316 | 0.02212068 |
| Tnrc6b | 1.654674946 | 0.03145674 |
| Sec14l4 | 1.65373874 | 0.00752421 |
| Meox2 | 1.653247439 | 0.00383536 |
| Pot1a | 1.652803063 | 0.00166244 |
| Cacna1h | 1.652598656 | 0.04213204 |
| Wdr35l | 1.651489961 | 0.02595636 |
| Ghrh | 1.650927386 | 0.00480585 |
| Lztfl1 | 1.648452138 | 0.0382769 |
| Ebf2 | 1.64810478 | 0.00982264 |
| Insig2 | 1.646778898 | 0.04623735 |
| Sppl2a | 1.646005093 | 0.03085522 |
| Fzd6 | 1.645413707 | 0.00062496 |
| Btg4 | 1.64427507 | 0.04062653 |
| Arl5b | 1.639867935 | 0.00718399 |
| Trim43a | 1.639802464 | 0.0369541 |
| Olr13 | 1.638874067 | 0.04918235 |
| Wnt10b | 1.635015372 | 0.00322807 |
| Gstt2 | 1.634936383 | 0.00279821 |
| Fpr1 | 1.634759567 | 0.03972246 |
| Zmym6 | 1.634153305 | 0.00457317 |
| Rfk | 1.633982652 | 0.01966642 |
| Ppp4c | 1.63147702 | 0.02849567 |
| Id2 | 1.630592744 | 0.04463848 |
| Add3 | 1.629059239 | 0.01902517 |
| Fcgr2b | 1.628518377 | 0.00020812 |
| Tcp11l2 | 1.628414907 | 0.00057173 |
| Reln | 1.628154567 | 0.04166278 |
| Ccdc28a | 1.627611448 | 0.00343924 |
| Stk17b | 1.626574986 | 0.0032331 |
| Idh1 | 1.625982241 | 0.0018791 |
| Slc22a6 | 1.625863642 | 0.01165981 |
| Nr4a2 | 1.624589284 | 0.03466725 |
| LOC680716 | 1.623953736 | 1.10E-05 |
| LOC678755 | 1.620820957 | 0.02664122 |
| Btk | 1.620048345 | 0.01856911 |
| Parvg | 1.619995868 | 0.0193018 |
| RGD1560151 | 1.618853837 | 0.00158089 |
| Gdf3 | 1.618308848 | 0.00377625 |
| Lrrn4cl | 1.617749614 | 0.01960542 |
| C4bpb | 1.617615882 | 0.03455086 |
| LOC499602 | 1.616951605 | 0.00164509 |
| Tubgcp5 | 1.616356017 | 0.02965049 |
| Cx3cr1 | 1.615835501 | 0.02356359 |
| Hspbap1 | 1.615302464 | 0.01558284 |
| Parp9 | 1.614796838 | 0.02872201 |
| RGD1562284 | 1.613764733 | 0.01219074 |
| Parvg | 1.613685316 | 0.03314576 |
| Pcdhgb7 | 1.613522766 | 0.00526266 |
| Sdccag8 | 1.612806621 | 0.04841235 |
| Sec3l1 | 1.611657556 | 0.04591215 |
| Kcnv2 | 1.610066972 | 0.01854041 |
| Nme7 | 1.609237579 | 0.01326328 |
| Dao | 1.607604848 | 0.02087738 |
| RGD1563866 | 1.607175043 | 0.0025552 |
| Mbp | 1.604662468 | 0.01840668 |
| Tnfsf18 | 1.604291051 | 0.01106355 |
| RGD1564171 | 1.603487381 | 0.03178238 |
| Gpr161 | 1.602770617 | 0.01135312 |
| Fcgr2a | 1.602535334 | 0.01532532 |
| Mfsd8 | 1.599998146 | 0.02126132 |
| Adh7 | 1.599812504 | 0.03480688 |
| Olr1169 | 1.599798865 | 0.03368697 |
| Fam181b | 1.599573405 | 0.00575193 |
| Ogt | 1.599276069 | 0.02432436 |
| Lcp2 | 1.598204107 | 0.03509472 |
| Rap1gap2 | 1.598000286 | 0.00394471 |
| Cd200 | 1.597976657 | 0.01397449 |
| Dnaaf1 | 1.597085482 | 0.02895265 |
| Cdh20 | 1.597049615 | 0.0354063 |
| LOC690217 | 1.596799676 | 0.00567386 |
| Ift88 | 1.596539964 | 0.01557125 |
| Ccnc | 1.594505787 | 0.02711037 |
| Fam178b | 1.593534477 | 0.0291169 |
| Npl | 1.592590106 | 0.00119949 |
| Chm | 1.591971822 | 0.0371008 |
| Zfp39 | 1.591918341 | 0.00280237 |
| RGD1311558 | 1.591587897 | 0.00370084 |
| Dedd | 1.591252558 | 0.00355245 |
| Capns2 | 1.590608994 | 0.02916487 |
| Mobkl2c | 1.590204785 | 0.02808207 |
| Serpini1 | 1.589898905 | 0.01724569 |
| Osap | 1.589898391 | 0.00932593 |
| Ctsc | 1.589342255 | 0.04656801 |
| LOC100365054 | 1.589194531 | 0.02451732 |
| Adam4l1 | 1.588256548 | 0.00094624 |
| LOC363337 | 1.588181725 | 0.02471755 |
| Tmem74 | 1.587865154 | 0.00277967 |
| RGD1310552 | 1.587500266 | 0.00908751 |
| Zfp322a | 1.586650493 | 0.02877164 |
| Rarg | 1.586570358 | 0.00818023 |
| RGD1560672 | 1.585519477 | 0.01560459 |
| Galc | 1.584795402 | 0.00182646 |
| Il22ra2 | 1.582365525 | 0.00833729 |
| LOC500035 | 1.581577224 | 0.03943929 |
| Bbs5 | 1.581348486 | 0.00109527 |
| Hbp1 | 1.581301354 | 0.01589601 |
| Klhl38 | 1.580417288 | 0.00493012 |
| MGC116121 | 1.580145053 | 0.0139541 |
| Gpr162 | 1.577561848 | 0.02008458 |
| Ube2v2 | 1.577510382 | 0.01086545 |
| Tmem165 | 1.577331249 | 0.0130657 |
| Lrrc23 | 1.577222212 | 0.00517588 |
| Chn2 | 1.577123969 | 0.0040608 |
| Slc13a3 | 1.57709183 | 0.0216026 |
| Scn2a1 | 1.576628581 | 0.02014614 |
| Fam46a | 1.5762882 | 0.02448433 |
| Ankrd28 | 1.575917124 | 0.00138002 |
| RGD1311251 | 1.575822712 | 0.01844263 |
| Pfkfb2 | 1.574954591 | 0.04230455 |
| RGD1306962 | 1.574197841 | 0.00256253 |
| Atp10d | 1.572333696 | 0.02595902 |
| Cpa6 | 1.57211643 | 0.00458628 |
| Atf2 | 1.570132598 | 0.03935829 |
| Rnasel | 1.567998088 | 0.01504224 |
| Zfp773-ps1 | 1.566701392 | 0.01535206 |
| Tnfaip6 | 1.565515725 | 0.0169809 |
| Stap1 | 1.564659573 | 0.00631603 |
| Cyb5d2 | 1.564647317 | 0.00644459 |
| LOC685020 | 1.56462635 | 0.00090409 |
| RGD1309823 | 1.564441342 | 0.00231512 |
| Pfkfb2 | 1.563190964 | 0.0132838 |
| Il17re | 1.562839185 | 0.02176445 |
| Spesp1 | 1.561846063 | 0.00238476 |
| LOC366300 | 1.560019973 | 0.04544649 |
| Cpne4 | 1.559476414 | 0.02063155 |
| Akr1c18 | 1.559213226 | 0.02170713 |
| RGD1562890 | 1.558999141 | 0.00029952 |
| Stk11 | 1.557927971 | 0.03823356 |
| Zfp426l | 1.557039992 | 0.00107468 |
| Tmed4 | 1.556224826 | 0.01884276 |
| Ccdc62 | 1.555728886 | 0.01690249 |
| Elovl7 | 1.555247262 | 0.00781269 |
| Slco4a1 | 1.554081143 | 0.03734018 |
| Abcc9 | 1.553869487 | 0.00101658 |
| Prkag2 | 1.550449461 | 0.04341608 |
| Tbc1d5 | 1.550254775 | 0.03729421 |
| Cyp2j3 | 1.549503342 | 0.01155091 |
| Pcmtd2 | 1.549343105 | 0.04182215 |
| Mterfd3 | 1.548153118 | 0.00126315 |
| Il1a | 1.547911046 | 0.01357018 |
| Tlr4 | 1.547592526 | 0.01997748 |
| Ncf1 | 1.54757143 | 0.01443885 |
| Per2 | 1.544837582 | 0.03033755 |
| Krt20 | 1.543881867 | 0.03629212 |
| Taf15 | 1.543688862 | 0.03051254 |
| Mtmr6 | 1.54302899 | 0.04239947 |
| Nfkbiz | 1.542343385 | 0.02038129 |
| Tmem68 | 1.542297985 | 0.03950486 |
| Comt | 1.542208687 | 0.02815186 |
| Actr6 | 1.540986618 | 0.03388179 |
| Slc16a4 | 1.540819856 | 0.00317972 |
| Cited2 | 1.540399436 | 0.04039958 |
| Fbxo8 | 1.539434872 | 0.00656135 |
| Fcgr2b | 1.538621635 | 0.03781841 |
| Fibin | 1.537907785 | 0.01256507 |
| Ift57 | 1.537796925 | 0.01652022 |
| LOC681994 | 1.537243564 | 0.04928874 |
| LOC681309 | 1.53680878 | 0.03229897 |
| Nqo2 | 1.535729367 | 0.000209 |
| Cd200 | 1.534928017 | 0.01871654 |
| RGD1309759 | 1.533669194 | 0.01955119 |
| Rbbp9 | 1.533555097 | 0.02199801 |
| Plk1s1 | 1.533160782 | 0.02341133 |
| Wars2 | 1.532904266 | 0.01976837 |
| Senp7 | 1.532275804 | 0.0116137 |
| Figf | 1.531455557 | 0.0148244 |
| Plcb4 | 1.53131714 | 0.02569808 |
| Ankrd34b | 1.531170988 | 0.01585665 |
| Fam35a | 1.530982614 | 0.0185899 |
| Tmod2 | 1.52981744 | 0.04678878 |
| Topors | 1.529170563 | 0.00471967 |
| Gnpda2 | 1.528824885 | 0.00607064 |
| RGD1562665 | 1.527238207 | 0.00210136 |
| Zcchc9 | 1.524399458 | 0.01755273 |
| Plekhm3 | 1.523639611 | 0.03489652 |
| Insr | 1.523525274 | 0.00111616 |
| Bmp4 | 1.522958539 | 0.02292433 |
| Znrf2 | 1.522853929 | 0.01114546 |
| Phox2a | 1.522759882 | 0.00050222 |
| Rnf32 | 1.52207857 | 0.04000133 |
| Dstn | 1.52123953 | 0.02617271 |
| RGD1565390 | 1.521114373 | 0.01705545 |
| Ufsp2 | 1.520876845 | 0.00749831 |
| LOC688507 | 1.520545794 | 0.0404728 |
| LOC499124 | 1.51835801 | 0.00356519 |
| RGD1306502 | 1.518353624 | 0.00663774 |
| RGD1359452 | 1.517911487 | 0.02716177 |
| Sfrp1 | 1.517316446 | 0.01538873 |
| Nudt6 | 1.515456912 | 0.01131198 |
| RGD1308818 | 1.515376591 | 0.03930195 |
| Alox5 | 1.5153462 | 0.00269991 |
| Mkks | 1.511775765 | 0.0036696 |
| Hmgcr | 1.511175272 | 0.04239102 |
| Lpar6 | 1.510471015 | 0.00091881 |
| Klhl7 | 1.509553791 | 0.00249525 |
| Gls2 | 1.509432769 | 0.01210942 |
| Oas1f | 1.507962655 | 0.00458683 |
| RGD1308065 | 1.507510935 | 0.01265228 |
| Spop | 1.507454719 | 0.03409651 |
| Mettl20 | 1.506936543 | 0.03039682 |
| Dnah10 | 1.506934002 | 0.03638265 |
| Klhdc8a | 1.50645276 | 0.00463617 |
| Dppa3 | 1.506355584 | 0.00950263 |
| Myh7 | 1.505812145 | 0.01981806 |
| Ccdc126 | 1.50488718 | 0.00140084 |
| Nipal2 | 1.504725159 | 0.01825858 |
| Polm | 1.504175601 | 0.01828992 |
| LOC681292 | 1.5039452 | 0.00423467 |
| Rnf14 | 1.502944776 | 0.0004902 |
| Wls | 1.502673248 | 0.0030828 |
| Cd59 | 1.502230818 | 0.00287246 |
| Adamts15 | 1.502175285 | 0.01556963 |
| Rmnd1 | 1.501787304 | 0.02198798 |
| Zpbp2 | 1.50122262 | 0.00683706 |
| Nmb | 1.501187622 | 0.03726775 |
| RGD1561667 | 1.500760678 | 0.00728668 |
| Sfrp4 | 1.500698854 | 0.01934797 |
| Abcc9 | 1.500356701 | 0.03091402 |
| Mylk2 | -19.86607833 | 0.00738372 |
| Gcg | -19.53277382 | 4.88E-05 |
| Iapp | -13.83746568 | 0.00015656 |
| Mylk2 | -11.23333864 | 0.01582342 |
| Ins2 | -10.36824014 | 0.0250099 |
| Slc2a2 | -9.561251042 | 0.00158921 |
| Ppy | -6.909947104 | 0.00039099 |
| Rbm24 | -6.628442269 | 0.01272383 |
| Inmt | -5.649246554 | 0.00987809 |
| Kbtbd5 | -5.425028563 | 0.03700539 |
| Tuba8 | -5.384037406 | 0.03172183 |
| Myoc | -5.018196625 | 0.01607292 |
| Asb16 | -4.6905396 | 0.0063106 |
| Cacng6 | -4.549753866 | 0.02243766 |
| Ins1 | -4.433926842 | 0.00803411 |
| Pcsk2 | -4.406858682 | 0.00102302 |
| Pitx2 | -4.405494199 | 0.03330077 |
| Calcr | -4.27898978 | 0.00206845 |
| Aspg | -4.221323712 | 0.00722401 |
| Entpd3 | -4.158331672 | 0.00037712 |
| Ucn3 | -4.099666915 | 0.03780645 |
| Grm4 | -4.063741218 | 0.04592993 |
| Fam151a | -3.968693826 | 0.0342755 |
| Egfl6 | -3.96074476 | 0.007298 |
| Myo18b | -3.902474486 | 0.03838821 |
| Ly6c | -3.746878943 | 0.00141211 |
| Abcb9 | -3.74341994 | 0.00018392 |
| Trdn | -3.67570818 | 0.00102665 |
| Ccng1 | -3.648761029 | 0.00023034 |
| Prkaca | -3.632561609 | 0.02056361 |
| Acsl6 | -3.580589014 | 0.02391929 |
| Scgn | -3.578994103 | 0.03920279 |
| Xcl1 | -3.572065991 | 0.00025833 |
| Scg2 | -3.384319796 | 0.02453552 |
| Ppy | -3.377243262 | 0.0346667 |
| Krt18 | -3.340317719 | 0.000407 |
| Olr1091 | -3.282314243 | 0.01582142 |
| Cilp | -3.165505715 | 0.02199429 |
| Rergl | -3.066241225 | 0.04261073 |
| Wnt4 | -3.051970675 | 0.03214913 |
| Pdlim7 | -3.042596527 | 0.02927897 |
| LOC683212 | -3.031758841 | 0.00730117 |
| Gnas | -3.021062575 | 0.00102429 |
| Pm20d2 | -2.990820444 | 0.0049315 |
| Olr154 | -2.985283476 | 0.00981376 |
| Tmem45b | -2.960591276 | 0.0370253 |
| Gck | -2.959523611 | 0.02198574 |
| Clcn1 | -2.940465234 | 0.03589985 |
| Tcrb | -2.894938099 | 0.04679035 |
| Smptb | -2.876454401 | 0.04211021 |
| Jag2 | -2.866243345 | 0.01818969 |
| Ly6g6f | -2.819995903 | 0.00102577 |
| Sim1 | -2.813480635 | 0.00838403 |
| Nppc | -2.783990523 | 4.63E-05 |
| Ttbk1 | -2.756596551 | 0.02007312 |
| Rbm20 | -2.735485737 | 0.00509943 |
| Olr181 | -2.732855666 | 0.04168514 |
| Ckap2l | -2.703992344 | 0.0443514 |
| Sostdc1 | -2.691753127 | 0.00230439 |
| LOC303341 | -2.676846601 | 0.0270521 |
| Fxyd6 | -2.651467381 | 0.04492992 |
| Ccdc64b | -2.620863166 | 0.01796152 |
| Car8 | -2.581827656 | 0.00858741 |
| Asb12 | -2.575957278 | 0.03517172 |
| LOC682419 | -2.537162106 | 0.04304863 |
| Olr1232 | -2.524924647 | 0.04410888 |
| Kcnk2 | -2.515850741 | 0.02454809 |
| Sox10 | -2.508770441 | 0.04236032 |
| LOC689352 | -2.484112251 | 0.00964346 |
| Adam24 | -2.460159068 | 0.01390708 |
| Ermap | -2.441377009 | 0.01746227 |
| Relt | -2.425918584 | 0.02661438 |
| Smyd5 | -2.40181073 | 0.0265685 |
| Grwd1 | -2.399401907 | 0.04087395 |
| Myh2 | -2.355046371 | 0.00853932 |
| Neurod1 | -2.346298228 | 0.03399832 |
| Extl1 | -2.340913134 | 0.00195151 |
| Chd5 | -2.318276158 | 0.04153978 |
| Trappc10 | -2.292674429 | 0.03930234 |
| Rem1 | -2.27085082 | 0.00588307 |
| Amy1a | -2.264522227 | 0.00014297 |
| Npy | -2.235740894 | 0.00595035 |
| Ly6g6c | -2.234553108 | 0.0370481 |
| Vars | -2.231542629 | 0.00528772 |
| Nes | -2.231529172 | 0.0278768 |
| Mttp | -2.230859054 | 0.00024314 |
| Rgs17 | -2.209699721 | 0.0092272 |
| Kcnn4 | -2.205807918 | 0.00205409 |
| Nkg7 | -2.19862349 | 0.00041122 |
| RGD1311564 | -2.194018768 | 0.00350037 |
| Tmem27 | -2.177692671 | 5.57E-05 |
| LOC502504 | -2.16766523 | 0.03381443 |
| LOC680711 | -2.166400785 | 0.03129039 |
| Cenpm | -2.160272688 | 0.00188875 |
| Accn1 | -2.155449856 | 3.78E-05 |
| Wnt5b | -2.150124352 | 0.01651683 |
| Tmem125 | -2.149561917 | 0.00336147 |
| Slc22a7 | -2.148633374 | 0.03409545 |
| Nnat | -2.146742771 | 0.04491394 |
| Slc38a1 | -2.141418197 | 0.02333445 |
| LOC300308 | -2.140083222 | 0.01131703 |
| Foxs1 | -2.135983249 | 0.00141458 |
| Lrtm2 | -2.129837606 | 0.01686954 |
| Ubtf | -2.12974406 | 0.00277781 |
| Fjx1 | -2.127641231 | 0.01993076 |
| Hip1r | -2.126948792 | 0.00166322 |
| Sh2b1 | -2.122533906 | 0.00341116 |
| Tp53i11 | -2.121771064 | 0.00158662 |
| Gys1 | -2.119728462 | 0.0123186 |
| Cenpt | -2.117858448 | 0.03396739 |
| Tex11 | -2.115262143 | 0.03583765 |
| Taar7d | -2.11521332 | 0.00275806 |
| Ldlrap1 | -2.106514578 | 0.00010775 |
| Kcnn4 | -2.105551505 | 0.00034374 |
| Ncoa4 | -2.103976611 | 0.00503577 |
| LOC367515 | -2.103083503 | 0.00944151 |
| LOC300024 | -2.10278935 | 0.00113347 |
| Slc26a2 | -2.098337051 | 0.04861207 |
| Amigo3 | -2.085919863 | 6.99E-05 |
| Cdh1 | -2.07927532 | 0.02313519 |
| Prf1 | -2.078090474 | 0.02335756 |
| Eln | -2.077505553 | 0.00208219 |
| Glis2 | -2.075531364 | 0.00273118 |
| Krt19 | -2.074149523 | 0.0175086 |
| Upk3bl | -2.053360257 | 0.00529284 |
| Pcdh9 | -2.051947188 | 0.00517704 |
| Mast2 | -2.03335808 | 0.00104544 |
| Slc27a3 | -2.029559872 | 0.00434969 |
| Tmie | -2.028487295 | 0.00227618 |
| Hoxd1 | -2.027519374 | 0.00538735 |
| Tnp1 | -2.020245332 | 0.00800763 |
| Xirp1 | -2.019018085 | 0.00105155 |
| Shh | -2.013807106 | 0.01166841 |
| Ms4a1 | -2.005025243 | 0.01699828 |
| Havcr2 | -2.003293772 | 0.00559087 |
| Npy | -1.991737241 | 0.00666718 |
| Wfdc15a | -1.989816097 | 0.00085134 |
| Rln1 | -1.988550959 | 7.18E-05 |
| Ddah1 | -1.986696278 | 0.00037713 |
| Aknad1 | -1.98620454 | 0.01038023 |
| Bglap | -1.985985652 | 0.0367353 |
| Lzts2 | -1.979343222 | 0.02789421 |
| Eno1 | -1.976599618 | 0.00731061 |
| Mat1a | -1.973248291 | 0.04732181 |
| Prss54 | -1.972985883 | 0.02521326 |
| RGD1305679 | -1.970427772 | 0.03561388 |
| Fam189a2 | -1.968717308 | 0.04486803 |
| Ubd | -1.963137286 | 0.00651923 |
| Emilin1 | -1.957748849 | 0.03130454 |
| LOC685125 | -1.94790214 | 0.00882166 |
| Tmem72 | -1.946553549 | 0.00270844 |
| Phldb3 | -1.945716566 | 0.03104165 |
| Sgsm2 | -1.945072324 | 0.01401752 |
| Xcl1 | -1.938005726 | 0.0043095 |
| Pdgfa | -1.937408263 | 0.0001101 |
| Cmtm2a | -1.93165092 | 0.03631368 |
| Slc17a9 | -1.930754187 | 0.00507229 |
| Ptprn | -1.930359207 | 0.00195177 |
| Cxcl9 | -1.925954873 | 0.0016048 |
| Micall2 | -1.923368238 | 0.0003217 |
| Eaf2 | -1.92141062 | 0.01472421 |
| Mrc2 | -1.918324122 | 0.00539724 |
| Snta1 | -1.918058248 | 0.02450834 |
| Kcnc2 | -1.916906984 | 0.04576268 |
| Orai1 | -1.915435041 | 0.02126745 |
| LOC686781 | -1.914866481 | 0.00388886 |
| Flnc | -1.914427466 | 0.0499139 |
| Gpr146 | -1.914313437 | 0.04795186 |
| Scx | -1.912476915 | 0.00469057 |
| Apoc4 | -1.912191131 | 0.00814078 |
| Mcm2 | -1.911716156 | 0.0036 |
| Tox3 | -1.901393412 | 0.04077473 |
| Bmp5 | -1.899330892 | 0.0215154 |
| Prx | -1.89548013 | 0.02793403 |
| Gpr152 | -1.887300347 | 0.01019869 |
| Sardh | -1.885520584 | 0.03064731 |
| Rnf187 | -1.884251272 | 0.00379515 |
| Prex1 | -1.878899542 | 0.03263609 |
| Grb7 | -1.876896939 | 0.00802855 |
| Lypd2 | -1.873863675 | 0.0277319 |
| Npepo | -1.873724702 | 0.00270783 |
| Dnajc30 | -1.871550097 | 0.0010229 |
| Bcar1 | -1.871204626 | 0.00392069 |
| Slc7a5 | -1.869623319 | 0.00102682 |
| Ddr1 | -1.868445738 | 0.01209596 |
| Dda1 | -1.863471171 | 0.01021069 |
| Tfeb | -1.863059349 | 0.03655421 |
| Sox6 | -1.862669826 | 0.00715371 |
| Ldlr | -1.854045394 | 0.01530554 |
| Rasa4 | -1.852516724 | 0.04838449 |
| Insl6 | -1.852240114 | 0.02640336 |
| Pla2g4c | -1.84975107 | 0.0349647 |
| Dhrs11 | -1.849252723 | 0.0273401 |
| P2ry2 | -1.846445483 | 0.00350997 |
| Tomm40 | -1.845228184 | 0.00105415 |
| Fscb | -1.844508324 | 0.00288571 |
| Rab3a | -1.844187615 | 0.02198938 |
| Cib3 | -1.841876887 | 0.00857878 |
| Slc44a4 | -1.836913595 | 0.04890664 |
| Cyp3a73 | -1.833000272 | 0.0437814 |
| Phlda1 | -1.830588006 | 0.02564437 |
| Plxdc1 | -1.826360378 | 0.03267523 |
| RT1-T24-1 | -1.824458498 | 0.00759807 |
| Esyt3 | -1.824032498 | 0.04598327 |
| RGD1564387 | -1.820802697 | 0.03426571 |
| Runx1 | -1.81827448 | 0.01180767 |
| Slc7a10 | -1.816596955 | 0.012551 |
| LOC100366132 | -1.816113456 | 0.01682687 |
| Map3k14 | -1.81039399 | 0.00833982 |
| Slc29a2 | -1.80989232 | 0.01743057 |
| Nelf | -1.808714833 | 0.04021463 |
| Zim1 | -1.805686955 | 0.04354269 |
| Bop1 | -1.804251349 | 0.01262549 |
| Rrad | -1.80160075 | 0.00646902 |
| Ctsw | -1.801312848 | 0.00200116 |
| RT1-CE13 | -1.798654389 | 0.01639649 |
| Htr5b | -1.796491073 | 0.00260326 |
| Clvs1 | -1.795365487 | 0.01031358 |
| Ttc36 | -1.79219154 | 0.04895512 |
| LOC100360737 | -1.788878206 | 0.01942459 |
| Clip2 | -1.787987727 | 0.01061333 |
| Abtb2 | -1.787955958 | 0.0021819 |
| Olr326 | -1.784772513 | 0.00892327 |
| Mamstr | -1.782859688 | 0.02118726 |
| Myo18a | -1.782743322 | 0.02796201 |
| Ppp1r1c | -1.782609871 | 0.04375664 |
| Cd5l | -1.781015987 | 0.03184899 |
| LOC498201 | -1.777005401 | 0.0129317 |
| Fam129b | -1.776889869 | 6.09E-05 |
| Jph2 | -1.770947119 | 0.01555353 |
| Ppm1f | -1.770052764 | 0.00075582 |
| Clcn2 | -1.769529034 | 0.00584083 |
| LOC500213 | -1.762425531 | 0.03542095 |
| Pgpep1 | -1.759671547 | 0.00040578 |
| Zbbx | -1.757238037 | 0.03614975 |
| Phlda3 | -1.75332183 | 0.0120323 |
| Fbn2 | -1.753043627 | 0.01326795 |
| Aldob | -1.750727403 | 0.00921973 |
| Git1 | -1.750301836 | 0.02593389 |
| RT1-T24-1 | -1.749547499 | 0.00129383 |
| Slc9a4 | -1.749257851 | 0.00209523 |
| RGD1564927 | -1.748719546 | 0.02800282 |
| Aspscr1 | -1.748569694 | 0.02181357 |
| Plxna3 | -1.747913511 | 0.03898866 |
| Polg | -1.747780406 | 0.00237596 |
| Kirrel3 | -1.747594495 | 0.02643302 |
| S1pr3 | -1.747237187 | 0.0153999 |
| Map1a | -1.744655512 | 0.03594275 |
| Cdh5 | -1.744282321 | 0.02111808 |
| Kcnd2 | -1.743736966 | 0.04942534 |
| Farp1 | -1.743700425 | 0.01255562 |
| Pcbp4 | -1.743173939 | 0.00371518 |
| Ctnnbip1 | -1.742729109 | 0.00936469 |
| Cog2 | -1.741510293 | 0.03717857 |
| Tbx2 | -1.740355862 | 0.01435577 |
| N4bp3 | -1.738926635 | 0.04841106 |
| LOC100359826 | -1.734213876 | 0.00970021 |
| RGD1564927 | -1.733441841 | 0.0111794 |
| Gipr | -1.731579669 | 0.02270916 |
| Nrgn | -1.731023287 | 0.04600984 |
| Slc29a4 | -1.728550437 | 0.01842702 |
| Cdc42ep1 | -1.727879529 | 0.02378528 |
| Acbd4 | -1.726957007 | 0.02014725 |
| Ube2v1 | -1.726955929 | 0.00280941 |
| Metrn | -1.726450854 | 0.02677462 |
| Colq | -1.726436494 | 0.00137793 |
| Arsi | -1.725050982 | 0.00044692 |
| Psd4 | -1.72465943 | 0.00615342 |
| Lrrn2 | -1.724541404 | 0.04291373 |
| Ddah1 | -1.723091085 | 0.00541216 |
| Kifc3 | -1.722253846 | 0.00084576 |
| Por | -1.722218272 | 0.02845331 |
| Trim25 | -1.720877814 | 0.00444546 |
| Flt1 | -1.71962541 | 0.01338302 |
| Fam64a | -1.719280731 | 0.00027343 |
| Nr2f6 | -1.718242386 | 0.00636007 |
| Tmem121 | -1.717894254 | 0.00401331 |
| Fmo5 | -1.716627006 | 0.00733639 |
| Scin | -1.715761511 | 0.01870993 |
| Cd180 | -1.71425643 | 0.00769139 |
| Zfp703 | -1.712993844 | 0.0026885 |
| Abcf2 | -1.711091528 | 0.00498991 |
| Kif4b | -1.711061403 | 0.02036462 |
| Cdt1 | -1.710138693 | 0.02237518 |
| Mul1 | -1.70848143 | 0.01237574 |
| Mybl2 | -1.708132631 | 0.03433808 |
| Glis2 | -1.707792861 | 0.01508492 |
| Olr308 | -1.705547774 | 0.04971621 |
| Rhox5 | -1.704931725 | 0.03796007 |
| Ehd1 | -1.703936612 | 0.00751184 |
| Grrp1 | -1.703432326 | 0.04933866 |
| Mdga1 | -1.703143741 | 0.04382732 |
| Gnb2 | -1.701808063 | 0.0040833 |
| Hdgf | -1.701267105 | 0.00429472 |
| Dbn1 | -1.698864945 | 0.00940521 |
| Irs3 | -1.695215245 | 0.016123 |
| Phldb1 | -1.693535818 | 0.00947861 |
| Hjurp | -1.69038605 | 0.00828966 |
| Bysl | -1.689234167 | 0.01413064 |
| Med29 | -1.688831235 | 0.00559389 |
| Astn1 | -1.688432378 | 0.00631177 |
| Dapk3 | -1.68727555 | 0.02003747 |
| Sorbs2 | -1.686225327 | 0.00540893 |
| Cd5l | -1.686139695 | 0.01936729 |
| Clec1b | -1.685144181 | 0.0369851 |
| Camk2b | -1.683396434 | 0.04137421 |
| RGD1304580 | -1.682009204 | 0.04102964 |
| Dnajb5 | -1.681418284 | 0.03071673 |
| Vasn | -1.679186337 | 0.00246967 |
| Psgb1 | -1.678533658 | 0.02634369 |
| LOC685297 | -1.677047789 | 0.04201238 |
| Tap1 | -1.676863242 | 0.02271971 |
| Plekhm2 | -1.675752632 | 0.00474527 |
| Plekha4 | -1.675354425 | 0.02519462 |
| Npy1r | -1.673805663 | 0.0472751 |
| Cfl1 | -1.671143166 | 0.00074046 |
| Olr20 | -1.670973631 | 0.01916954 |
| Tnk2 | -1.66826048 | 0.00069448 |
| Med16 | -1.667750183 | 0.02552785 |
| Tmem150c | -1.66729721 | 0.03355577 |
| Urgcp | -1.664554569 | 0.00741529 |
| Cdk16 | -1.663794938 | 0.01125037 |
| Mxd3 | -1.663345576 | 0.00897206 |
| Paqr5 | -1.662854648 | 0.00109242 |
| RGD1306091 | -1.662807661 | 0.03990111 |
| Dlk2 | -1.660525257 | 0.00596606 |
| Tsta3 | -1.660144515 | 0.00153472 |
| Dusp10 | -1.659606984 | 0.04974571 |
| Sox11 | -1.659583862 | 0.00024991 |
| Zbp1 | -1.657836617 | 0.03225161 |
| Mapk12 | -1.657813213 | 0.03304433 |
| Tgfb1 | -1.655132062 | 0.02976371 |
| Gzmk | -1.652712523 | 0.00341167 |
| Tmem120a | -1.650852243 | 0.00748187 |
| Lman1l | -1.650792246 | 0.04287976 |
| LOC680531 | -1.650452746 | 0.02040792 |
| Vom1r55 | -1.649110419 | 0.03706828 |
| Rcn3 | -1.649068697 | 0.00925655 |
| Samd4a | -1.64754667 | 0.01138799 |
| Btbd2 | -1.647480132 | 0.00774055 |
| Gldn | -1.647473394 | 0.00610671 |
| Rab27b | -1.646540045 | 0.00175008 |
| Ddx49 | -1.644514999 | 0.01190614 |
| Abl1 | -1.644230051 | 0.0040257 |
| Ccdc88b | -1.643415524 | 0.01343735 |
| Pola2 | -1.642514758 | 0.02546761 |
| Lrit1 | -1.641821897 | 0.03113433 |
| Akt2 | -1.640757544 | 0.01169703 |
| Pacrg | -1.636611544 | 0.00838813 |
| Ccdc106 | -1.636239537 | 0.01009842 |
| Haus8 | -1.634937894 | 0.04197969 |
| Rtkn | -1.6322228 | 0.00947065 |
| Oasl | -1.630823819 | 0.00543539 |
| Capn13 | -1.630185531 | 0.02142468 |
| Foxi2 | -1.626739491 | 0.00308691 |
| Nsun5 | -1.625224555 | 0.00172975 |
| Dkc1 | -1.623956138 | 0.02153195 |
| Fam38a | -1.623366859 | 0.0078917 |
| Foxp4 | -1.621354242 | 0.01895824 |
| Ptges2 | -1.621155373 | 0.03034376 |
| Abhd2 | -1.620225292 | 0.01101168 |
| Fzd2 | -1.619979624 | 0.01228619 |
| Aes | -1.618808878 | 0.02361964 |
| Tnfrsf12a | -1.617760828 | 0.0022992 |
| Zfp703 | -1.617161392 | 0.00602052 |
| Skiv2l | -1.6167182 | 0.00838787 |
| Cenpb | -1.616458348 | 0.00576261 |
| Lyg2 | -1.615334448 | 0.0203621 |
| RGD1564915 | -1.614341462 | 0.04880098 |
| Suv39h1 | -1.613448319 | 0.01160774 |
| Ube2v1 | -1.611825988 | 0.00461917 |
| Rangrf | -1.610491262 | 0.0369997 |
| Ube2v1 | -1.609334253 | 0.00846838 |
| Rhpn1 | -1.608976439 | 0.04814778 |
| Ckap4 | -1.608730134 | 0.00380407 |
| Slc35e1 | -1.607965255 | 0.01062053 |
| Mmp15 | -1.607753244 | 0.02784011 |
| Med22 | -1.606842806 | 0.04830424 |
| Anapc1 | -1.605220998 | 0.01228871 |
| Arhgap22 | -1.603657813 | 0.00153648 |
| LOC679127 | -1.603240657 | 0.01017951 |
| Usp36 | -1.603206986 | 0.01539802 |
| RGD1310727 | -1.602737881 | 0.00720413 |
| Pum1 | -1.601890759 | 0.01080926 |
| Olr1002 | -1.601856191 | 0.0307328 |
| Tcfap2a | -1.600815824 | 0.0342535 |
| RGD1565693 | -1.600793262 | 0.00755137 |
| Hist2h3c | -1.600283859 | 0.01602628 |
| RGD1309543 | -1.59932854 | 0.00816633 |
| Pmf1 | -1.599074439 | 0.01808114 |
| Gp1bb | -1.598679604 | 0.01832964 |
| LOC690422 | -1.598197682 | 0.00840227 |
| Fam184b | -1.597673342 | 0.04485514 |
| Ptbp1 | -1.595415832 | 0.00485797 |
| Plaur | -1.593033935 | 0.03019742 |
| Arhgef40 | -1.591934341 | 0.00573014 |
| Spsb1 | -1.589758696 | 4.25E-05 |
| RGD1562846 | -1.589725858 | 0.00084943 |
| Plaur | -1.589497815 | 0.01842374 |
| LOC685685 | -1.587874509 | 0.04367034 |
| Upk1b | -1.58720631 | 0.00124233 |
| Gpsm1 | -1.587018156 | 0.0478123 |
| Aox4 | -1.586493159 | 0.02951474 |
| Plekhh3 | -1.584663112 | 0.02052062 |
| Cstf1 | -1.582492505 | 0.00921422 |
| Cd99 | -1.582157255 | 0.00111049 |
| Hspbp1 | -1.580612512 | 0.00553605 |
| Cnp | -1.578647612 | 0.04047143 |
| Fiz1 | -1.577789855 | 0.01240434 |
| LOC686323 | -1.577706668 | 0.00564602 |
| Dcaf15 | -1.576712805 | 0.01214734 |
| Ino80d | -1.57642795 | 0.03187999 |
| Ppp1r16a | -1.57506187 | 0.03486421 |
| Gna12 | -1.574349264 | 9.17E-05 |
| Adamtsl2 | -1.574316199 | 0.04340099 |
| Pyy | -1.574011957 | 0.03328294 |
| Sphk1 | -1.573715845 | 0.0042739 |
| Wdr18 | -1.573427786 | 0.00831397 |
| Arse | -1.573149231 | 0.03973351 |
| Tsc22d4 | -1.570690141 | 0.02736409 |
| Pdxk | -1.570516609 | 0.00624158 |
| Plekhg5 | -1.570432462 | 0.00740684 |
| Mettl11a | -1.568721121 | 0.00245327 |
| Tp53i13 | -1.56850921 | 0.0085552 |
| Ccdc85b | -1.566628201 | 0.01345488 |
| Trex1 | -1.566510022 | 0.04407841 |
| Wasl | -1.566247348 | 0.02381827 |
| Ube2v2 | -1.566229001 | 0.00617557 |
| LOC687399 | -1.564851367 | 0.02558717 |
| Fem1a | -1.564374582 | 0.02092925 |
| Gdf5 | -1.563571941 | 0.03721892 |
| Rcor2 | -1.563095581 | 0.0067508 |
| LOC683674 | -1.562246961 | 0.03001698 |
| Zfp668 | -1.562130882 | 0.00859361 |
| C1qtnf4 | -1.561568367 | 0.01718611 |
| Usp51 | -1.561499492 | 0.04127541 |
| Expi | -1.561425064 | 0.04024632 |
| Tk1 | -1.560371983 | 0.00470521 |
| Rab1b | -1.559888453 | 0.01825909 |
| Irf1 | -1.559399237 | 0.00723687 |
| Ints5 | -1.558079916 | 0.0099773 |
| Pc | -1.557851626 | 0.02498148 |
| Gramd4 | -1.556968223 | 0.01179461 |
| Rcan1 | -1.556682331 | 0.00760022 |
| Rfng | -1.555356361 | 0.0078603 |
| Ifng | -1.555019387 | 0.00137222 |
| Zfp384 | -1.553537464 | 0.03289781 |
| Cops7b | -1.553353948 | 0.00056296 |
| Preb | -1.553163813 | 0.00418424 |
| Vps72 | -1.552859891 | 0.00021457 |
| Ccnd1 | -1.55274921 | 0.0175202 |
| Cabin1 | -1.552024322 | 0.01106539 |
| Tanc2 | -1.550822531 | 0.00074535 |
| Tnks1bp1 | -1.550533289 | 0.01267491 |
| Tbrg4 | -1.550206063 | 0.00715169 |
| Zfp335 | -1.549647055 | 0.00585044 |
| Chtf18 | -1.548516154 | 0.00169059 |
| Jund | -1.547030171 | 0.00848237 |
| Sf3b4 | -1.546985134 | 0.02148153 |
| Unc119b | -1.546362617 | 0.00756465 |
| Foxk1 | -1.543726313 | 0.0460934 |
| Myo1c | -1.542975335 | 0.01042307 |
| Slc7a1 | -1.540846343 | 0.00232656 |
| Itpripl2 | -1.540773897 | 0.03211927 |
| Kifc1 | -1.540352991 | 0.0026285 |
| Smad7 | -1.54005798 | 0.02541784 |
| Scx | -1.540004607 | 0.00333911 |
| Lamb3 | -1.539669073 | 0.00722443 |
| Slc44a2 | -1.538966577 | 0.03571106 |
| Rcc2 | -1.538132123 | 0.02898144 |
| Rangap1 | -1.537363157 | 0.00626795 |
| Oasl2 | -1.535603053 | 0.01135338 |
| Klf16 | -1.535222932 | 0.03917112 |
| RGD1311273 | -1.534834714 | 0.0350441 |
| Selm | -1.532893181 | 0.00741088 |
| Ifrd2 | -1.530440545 | 0.01636254 |
| B3gat3 | -1.530030063 | 0.0346584 |
| Mapk11 | -1.529873889 | 0.04467946 |
| Fam54b | -1.529847838 | 0.03227701 |
| LOC679462 | -1.52709061 | 0.04672779 |
| Dgcr6 | -1.526377912 | 0.0346956 |
| Pxn | -1.52575202 | 0.00131927 |
| Pcdh12 | -1.525410075 | 0.00762674 |
| Fgd1 | -1.524890836 | 0.03178128 |
| Il3ra | -1.524528548 | 0.03374753 |
| Nle1 | -1.524284817 | 0.02307763 |
| Sepw1 | -1.524227412 | 0.01080635 |
| Bcorl1 | -1.524089367 | 0.01075935 |
| Cnpy3 | -1.524018765 | 0.031302 |
| Npepl1 | -1.523235493 | 0.00721951 |
| RGD1564927 | -1.522895906 | 0.03940934 |
| Thra | -1.522200079 | 0.01316018 |
| Arf6 | -1.521109312 | 0.01120282 |
| Ube2ql1 | -1.520821782 | 0.02197612 |
| Ncam1 | -1.520643183 | 0.02438239 |
| Mcm5 | -1.520426771 | 0.00728376 |
| Prrc2a | -1.520199151 | 0.01975256 |
| Lepre1 | -1.519156324 | 0.03412838 |
| Usp51 | -1.518821332 | 0.01383065 |
| Pdha2 | -1.518791679 | 0.03295447 |
| Ptpn23 | -1.517749818 | 0.04105925 |
| Troap | -1.515768923 | 0.00604216 |
| Dusp3 | -1.515654266 | 0.01352688 |
| Ager | -1.514763504 | 0.00905199 |
| Pde1c | -1.514284695 | 0.04918602 |
| Osbpl5 | -1.513337917 | 0.0417117 |
| Mpst | -1.510615156 | 0.01363039 |
| Grk6 | -1.509669696 | 0.01509652 |
| Armc6 | -1.507400072 | 0.01983801 |
| Rpsa | -1.506531495 | 0.0024471 |
| Lrfn4 | -1.506529406 | 0.00490034 |
| Hist1h1d | -1.50614218 | 0.00168804 |
| Gpam | -1.505437068 | 0.01094375 |
| Tp53 | -1.504605219 | 0.00353386 |
| Dbn1 | -1.504225299 | 0.02456749 |
| Dlgap4 | -1.503739329 | 0.01640597 |
| Pemt | -1.503696977 | 0.02738623 |
| Tmem82 | -1.502866438 | 0.02911042 |
| Acy1 | -1.502494038 | 0.01032657 |
| Chmp1a | -1.50207946 | 0.00916303 |
| Vwa3b | -1.502032053 | 0.03501687 |
| Wwp2 | -1.500853333 | 0.00183317 |
| Pkia | -1.500706032 | 0.0252812 |
| Gab2 | -1.500604615 | 0.02106387 |
| Pex6 | -1.500277145 | 0.02511312 |
